# Effects of different stocking densities on growth, nutritional quality, stress and antioxidant response in *Labeo rohita*; cultured in in-pond raceway system

**DOI:** 10.1371/journal.pone.0298753

**Published:** 2024-05-24

**Authors:** Qandeel Minahal, Shafaq Fatima, Wajeeha Komal, Razia Liaqat

**Affiliations:** 1 Faculty of Natural Sciences, Department of Zoology, Lahore College for Women University, Punjab, Pakistan; 2 Department of Biological Sciences, Purdue University Fort Wayne, Fort Wayne, Indiana, United States of America; Tamil Nadu Dr J Jayalalithaa Fisheries University, INDIA

## Abstract

A 171-day long experimental trial was undertaken to study intricate physiological response of rohu (*Labeo rohita*) under stress caused by high stocking density in In-pond raceways system (IPRS). Fingerlings of rohu (initial body weight: 250 ± 1.20 g) were cultured at three different stocking densities; low density (LD) (2.27 kg/m^3^), medium density (MD) (3.79 kg/m^3^) and high density (HD) (5.30 kg/m^3^) in raceways of IPRS production system. Each treatment was in triplicate. Fish growth exhibited a decline in HD treatment statistically as its average weight gain/fish/day was 4.21 g as compared to MD (4.82 g) and LD (4.74 g). Nutritional profile of rohu indicated by the content of crude protein, fatty acids, and profile of amino acids was up to the set dietary benchmarks. Survival rate of fish in all the treatment groups was greater than 99%. The elevated cortisol levels observed in the HD treatment in contrast to the other treatments suggested the presence of stress. The levels of superoxide dismutase, catalase and glutathione peroxidase were also higher in HD as compared to other treatments. However, there were no difference in the level of MDA between the three treatments. Activity of amylase, protease was significantly different in treatment whereas the difference in lipase activity was found to be insignificant. It can be concluded that medium stocking density i.e. 3.79 kg/m^3^ outperformed the high density (5.30 kg/m^3^) in different aspects of this study. Nevertheless, additional research is imperative to ascertain whether any intermediate stocking density between medium (3.79 kg/m^3^) and high (5.30 kg/m^3^) such as 4 kg/m^3^, 4.5 kg/m^3^, or 5 kg/m^3^, could potentially serve as suitable options for rohu. It is also suggested that brood stock of rohu should be genetically improved to obtain stress resilient fingerlings which will perform better at high stocking density at large scale production level.

## 1. Introduction

Aquaculture, a multimillion commercial enterprise, has emerged as a vital agricultural sector worldwide. Given the current state of food security, traditional farming methods alone cannot be relied upon in Pakistan and other developing countries [1]. To meet the future per capita fish consumption, according to current per capita consumption i.e 1.90 kg/year, aquaculture production will increase from 178 million tonnes (2020) to 278 million tonnes by 2030 [2]. To achieve this, the integration of intensive systems is imperative, leveraging modern expertise in pond construction, feed formulation, and stocking densities [3]. IPRS technology is a modern intensive culture technology, combination of recirculating aquaculture system, pond culture, raceways, and cage culture to enhance the production of fish. The In-Pond Raceway System encounters certain challenges, such as susceptibility to disease outbreaks resulting from high densities and the necessity for alternative electrical power sources. Nevertheless, the IPRS facilitates improved application of preventive measures for maintaining fish health, thereby enabling increased annual yields from the pond. Fingerlings acquired from genetically improved brood stock can improve the disease resistance thus making IPRS better option for farmers [4]. This technology has been successfully used in several countries including Pakistan [5] and utilized in present study as well. However, in future this technology can be modified by altering the design and construction materials to render IPRS technology economically viable for small and medium-scale farmers.

Crowding stress is one of the most common and potentially harmful factors that negatively affect fish health, growth and productivity [6]. Reduced weight gain in fish can be attributed to inadequate feeding practices, encompassing both instances of either being underfed or fish cease feed intake under condition of stress. In case of high stocking density, fish appetite for food is reduced as an adaptation to stress and competition to survive in a restricted environment. During the exposure to stress, accelerated metabolism has been observed in fish indicated by hyperglycemia, as plasma glucose levels correlates with metabolic rate positively such as observed in largemouth bass (*Micropterus salmoides*). Consequently, fish necessitate augmented energy resources to effectively manage the stressors, thereby potentially impeding optimal growth performance. [7]. Hematological and biochemical parameters are frequently employed as indicators of fish physiological status. Hemoglobin (Hb) is linked with stress responses as its synthesis within the erythrocytes alters in response to heightened adrenalin production during stress. The increased production of RBC and Hb serves to uphold optimal levels of oxygen within the blood stream, facilitating its efficient transport to various tissues as reported in Amur sturgeon [8]. Concurrently, the levels of triglycerides experience a reduction, attributed to their metabolic conversion into simple sugar (glucose) to meet heightened energy requirements [9].

Typically, the indicators of fish stress are levels of glucose and cortisol [10,11]. Elevation in glucose level indicates the mobilization of gluconeogenesis and glycogenolysis pathways to meet the increasing energy demand in density induced stress [8]. In a stressful situation, hypothalamus-pituitary-inter renal axis secretes the principal glucocorticoid, namely cortisol. This increased level of cortisol within plasma helps to satisfy heightened energy needs, eliciting the upregulation of glucogenesis and hepatic glycogenolysis pathways to escalate glucose production [8,9,11]. Concomitantly, stress conditions incite a surge in intracellular reactive oxygen species (ROS) production causing oxidative stress [12]. Fish subjected to chronic crowding stress experience modifications in the activities of antioxidants such as superoxide dismutase (SOD), catalase (CAT), glutathione peroxidase (GPx) and malondialdehyde (MDA) [13]. Increase in activity of these biomarkers suggests the activation of a potential protective mechanism that becomes operational to mitigate the presence of these ROS [14]. This study focuses on assessing the levels of glucose, cortisol and all these four antioxidants to find out whether the factor of stocking density has caused stress or not.

The total freshwater aquaculture production in Pakistan is 200,003 MT per year and cyprinids contribute approximately 77% in this production. Within the diverse array of fish species under cultivation, rohu is the fifth largely cultured specie in the world [15]. Rohu is the prominent and common fish stocked greatly due to its broader feeding niche [16]. Rohu requires 30% protein in its diet [17] This study holds its novelty due to distinct intensive rearing conditions that have never been used earlier for the culturing of rohu. The maximum stocking density in which rohu has been reared in previous studies is 0.246 kg/m^3^ in biofloc system [18],1.16 kg/m^3^ in aquarium [19], 0.41 kg/m^3^ in cages [20] and 0.91 kg/m^3^ in floating cages [21]. In addition to this, no reported literature is available on culturing of rohu at large commercial scale to investigate the most optimum stocking density in intensive culture. The primary objective of this investigation was to determine the stocking density at which rohu performed better, considering all aspects such as growth, production, nutritional content, stress tolerance, and defense system. A comprehensive examination is imperative to integrate the IPRS technology with the existing earthen pond system, aiming to augment aquaculture production.

## 2. Materials and methods

### 2.1. IPRS system

**Fig 1:**
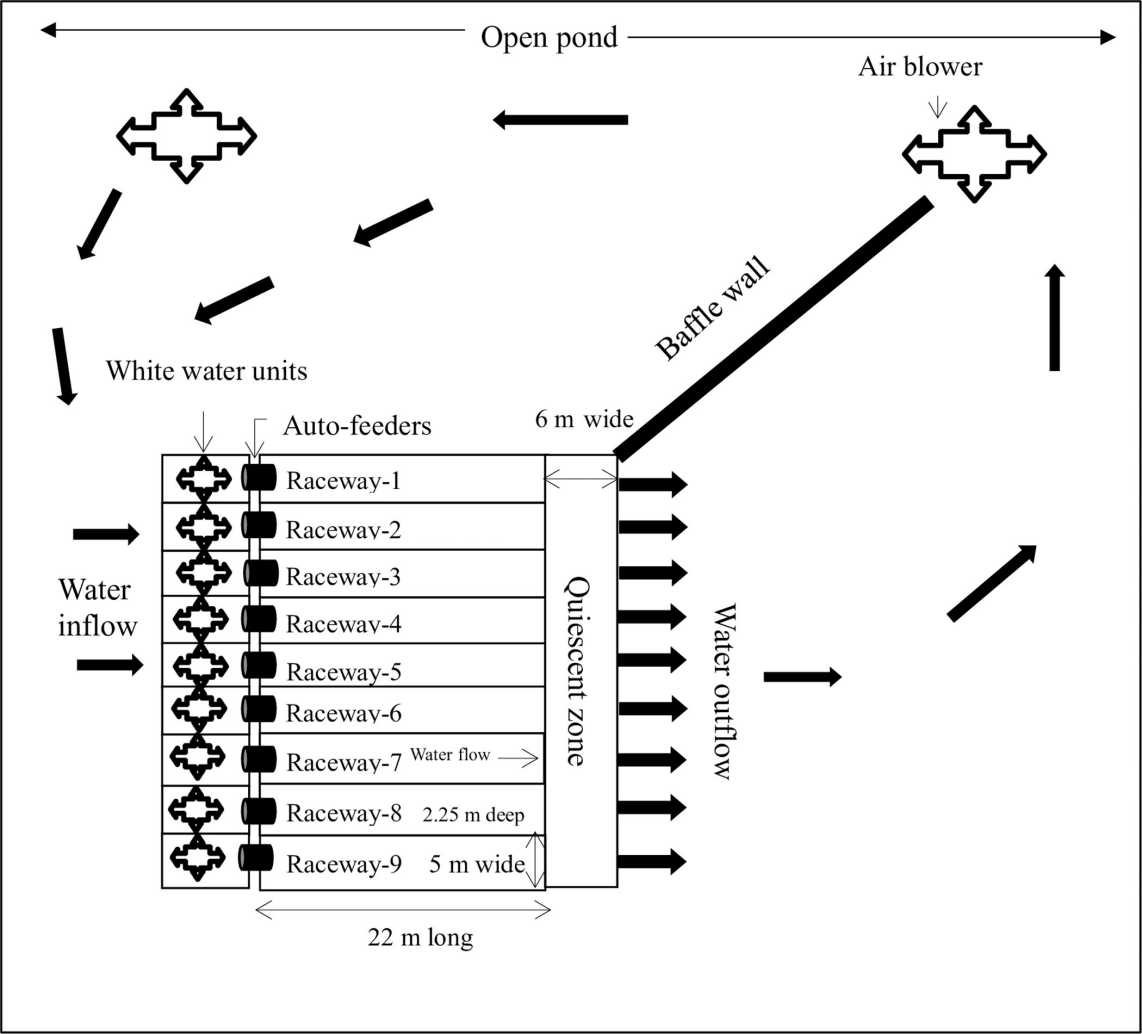
A sketch showing the IPRS production system utilized in the current study. The dimensions of the raceways can be observed in raceway eight and nine of the image. Each raceway is equipped with fixed auto feeder. Arrows within the image indicate the direction of water flow, illustrating the movement from the White-Water Units towards the Quiescent Zone.

The IPRS system was constructed within a twelve-acre earthen pond, supplied with well water having a pH of 7.80 and a total dissolved solids (TDS) level of 352 parts per million. The IPRS system comprised of nine individual raceways constructed according to the specifications outlined by [22] (Fig 1). Each raceway’s land area measured 22 meters in length, 5 meters in width, and 2.25 meters in depth. These raceways were interconnected, sharing a common Quiescent Zone (QZ) measuring 3 meters in length, 15.61 meters in width, and 2.35 meters in depth, alongside a sludge collection unit measuring 22 meters in length, 2 meters in width, and 2.5 meters in depth. To confine the fish within each raceway, an end partition barrier spanning the width of each unit was employed. An imported five-meter-long airlift system, consisting of four diffuser grid racks (each 1.2 meters long and 1.05 meters wide) along with deflection hoods for each raceway, was assembled based on the specifications detailed by Chappell (2017) and sourced from Xuan Cheng Dingxing Environmental Protection Engineering Co., Ltd., China.

Aeration for each raceway was provided by a 2.2 kW ring blower capable of delivering a maximum airflow of 210 cubic meters per hour directly into the water column via two diffuser grid airlifts. The diffuser grids, constructed using anti-microbial tubing with outer and inner diameters of 25.4mm and 12.7mm respectively, facilitated an airflow of 2.2 cubic meters per hour per meter. Each raceway could sustain a suggested maximum airflow of 88 cubic meters per hour with two diffuser grids. Additionally, bottom aeration for each raceway was facilitated by a 3-kW ring blower capable of delivering an airflow of 230 cubic meters per hour, distributed through 16 pieces of anti-microbial tubing, each one meter in length, with an airflow of 2.2 cubic meters per hour.

Furthermore, an earthen baffle wall was built at end of the system, extending 120 meters into the pond. Its purpose was to guide water flow within the pond, preventing the short-circuiting of return flow to the raceways. In the Quiescent Zone (QZ), a continually oscillating vacuum head measuring 2.7 meters long was connected to collect waste material from the bottom of the QZ and transfer it to waste collection units. Each raceway was equipped with an automatic feed delivery system capable of storing up to 100 kilograms of feed. This system included a 3–20-meter feed-casting distance, a 16W vibration motor, and a maximum feeding capacity of 150 kilograms per hour. To ensure continuous operation, power backup was provided by two generators with capacities of 20 kW and 30 kW in the event of a power failure, ensuring the functionality of the entire system including aeration and feeding. Regular system and data maintenance activities such as daily water quality data collection, daily cleaning of probe membranes of water quality meter, monthly probe calibration, daily mortality checks, daily removal of mortalities, and biweekly cleaning of the feed delivery system were conducted on a routine basis.

### 2.2. Experimental design

This study was commenced after ethical approval from Lahore College for Women University (Zoo/LCWU/930). Fingerlings (initial weight: 250.00±10.25 g; age: 90 days) for this study were procured from a local fish hatchery. The experimental design consisted of three treatments (Low density (LD), Medium density (MD), High density (HD)) while each treatment has three replicates (three raceways per treatment). The number of fingerlings stocked in each replicate (raceway) for different treatment was; 6,000 fingerlings/replicate (2.27 kg/m^3^) in LD, 10,000/replicate (3.79 kg/m^3^) in MD and 14,000/replicate (5.30 kg/m^3^) in HD. Collectively, nine raceways were used to study three different treatments, each in triplicates. Total number of fingerlings stocked in three raceways for each treatment was; 18,000 in LD, 30,000 in MD and 42,000 in HD treatment (Table 1). Trial period in this study was 171 days (June-November).

**Table 1:** Summary of growth parameters in low density (LD), medium density (MD), and high density (HD) treatments.

#	Group	Stocking Density		Initial Biomass			Survival Rate %	Harvested Biomass			Biomass Gained (kg)		Wt. Gain/ Day/ Fish (g)	FCR
		No.	Fish/m^3^	Avg. I. Wt. (g)	Total Biomass (kg)	kg/m^3^		Avg. F. Wt. (g)	Total Biomass (kg)	kg/m^3^	Total Weight Gain	Total Weight Gain/Day		
	**Single Raceway**													
**1**	LD	6,000	27	250.00	1,500.00	2.27	99.92	1,060	6,355	28.89	4,855	28.39	4.74	1.90
**2**	MD	10,000	46	250.00	2,500.00	3.79	99.91	1,075	10,740	48.82	8,240	48.19	4.82	1.89
**3**	HD	14,000	64	250.00	3,500.00	5.30	99.92	970	13,569	61.68	10,069	58.88	4.21	1.88
	**Three Raceways**													
**1**	LD	18,000	27	250.00	4,500.00	6.82	99.92	1,060	19,064	28.89	14,564	28.39	4.74	1.90
**2**	MD	30,000	46	250.00	7,500.00	11.36	99.91	1,075	32,221	48.82	24,721	48.19	4.82	1.89
**3**	HD	42,000	64	250.00	10,500.00	15.91	99.92	970	40,708	61.68	30,208	58.88	4.21	1.88

Avg. I. Wt.: Average initial weight; Avg. F. Wt.: Average final weight.

Fish in all treatments were fed with commercial floating feed (Protein: 30.43±0.28%, Starch: 23.00±0.34%, Moisture: 11.17±0.14%, Ash: 7.40±0.34%, Fat: 5.67±0.08%, Fiber: 3.97±0.14%) at the rate of 2% of total biomass on daily basis. Random weight check of fish was performed on monthly basis in each treatment. Parameters of water quality such as water temperature (°C), pH, dissolved oxygen (DO) (mg/L), ammonia (ppm) and nitrites (mg/L) were noted daily (at 0.15 m and 1.22 m depth in raceways) at interval of four hours from 8:00 AM until 12:00 AM. Detailed information of water quality parameters has been provided in supporting data (S1 Table in S1 File).

### 2.3. Sampling and analysis

From each replicate of one treatment, 20 fish were randomly samples after every 30 days of the trial. Collectively, sixty fish were samples from each treatment. Fish were anesthetized using clove oil (0.8 ml/L) (Sigma-Aldrich). The total body weight and total body length of each fish was measured. Blood specimens were collected from the caudal vein and subsequently transferred to EDTA coated tubes. A portion of the blood sample was used to measure hematological parameters. While rest of the blood was then subjected to centrifugation at 5,000 rpm for a duration of 15 minutes. Following centrifugation, the plasma fraction was isolated and stored in Eppendorf tubes to facilitate subsequent analytical investigation.

Muscle samples were collected and stored at -20°C until different analysis were performed to assess its nutritional quality such as proximate analysis, fatty acids and amino acids analysis, following the guidelines of Association of Official Analytical Chemists (AOAC**)** (2005). The intestinal samples were first weighed and then mixed in 0.86% sterile normal saline solution at a ratio of 1 part tissue to 9 parts Salines, using a high-speed tissue homogenizer. Subsequently, the resulting mixtures were subjected to centrifugation at 5000 revolutions per minute for 15 minutes, after which the resulting liquids (supernatants) were gathered and kept at a temperature of -20°C for subsequent analysis. All analysis were done in triplicate. Different growth parameters were calculated by using the given formula:

$$Weight\;gain\;(\%)=\frac{Final\;weight-Initial\;weight}{Initial\;weight}\times 100$$

$$Condition\;factor\;(\%)=\frac{Total\;body\;weight}{Total\;body\;length3}\times 100$$

$$Specific\;growth\;rate\;(\%)=\frac{Ln\;final\;weight-Ln\;initial\;weight}{Time\;interval\;in\;days}\times 100$$

$$Feed\;conversion\;ratio=\frac{Weight\;of\;feed\;consumed}{Weight\;gain\;(wet\;weight)}$$

$$Condition\;factor\;(\%)=\frac{Total\;body\;weight}{Total\;body\;length3}\times 100$$

$$Survival\;rate\;(\%)=\frac{Final\;number\;of\;fish}{Initial\;stocking\;density}\times 100$$

$$Hepatosomatic\;index=\frac{Liver\;weight}{Total\;body\;weight}\times 100$$

### 2.4. Blood biochemistry and hematology

Plasma separated after centrifugation was transferred to separate Eppendorf tubes. Triglyceride (TG) (mg/dl) was estimated through a triglyceride colorimetric assay kit (Thermo Fisher Scientific, USA, CAT No. EEA028) following the protocols of the manufacturer. The level of albumin (Alb) (g/dl) was determined through the use of bromocresol green (BCG) dye binding technique, utilizing an albumin kit (LOT. DR379E249; ANMOL-LAB Pvt. Ltd, India). The quantification of alkaline phosphatase (ALP) (Unit/L) (U/L) was carried out using commercial kit (Thermo Fisher Scientific, USA, CAT No. EEA002, E.C. 3.I.3.1.). Aspartate aminotransferase (AST) (U/L) was estimated through commercial ELISA kit (Thermo Fisher Scientific, USA, CAT No. MAK055, E.C. 2.6.1.1.). Activity of alanine aminotransferase (ALT) (U/L) was measured using commercial ELISA kit (Thermo Fisher Scientific, USA, CAT No. MAK052, E.C. 2.6.1.2.). The concentration of glucose (GLU) (mg/dl) was measured by using laboratory blood glucose analyzer.

Hematological parameters such as hemoglobin (Hb) (g/dl), white blood cells (WBC) (103/μL) count such as neutrophils (%), eosinophils (%), lymphocytes (%) and monocytes (%), red blood cell (RBC) (106/μL) count, the content of hematocrit (HCT) (%), platelets (103/μL), mean corpuscular volume (MCV) (FL), mean corpuscular hemoglobin concentration (MCHC) (grams/deciliter) and mean corpuscular hemoglobin (MCH) (pg) was determined through automated blood analyzer (Sysmex-KX-21, Japan).

### 2.5. Digestive enzymes assay

For digestive enzyme analysis, the supernatant of processed whole intestine samples was utilized. Activity of lipase (U/L) was assayed with a commercial ELISA kit (Sigma Aldrich, USA, CAT No. MAK046, EC 3.1.1.3) with a detection limit of 5 U/L to 250 U/L at 37°C and 570 nm of wavelength. Amylase (U/L) activity was measured using a commercial ELISA kit (Sigma Aldrich, USA, CAT No. MAK009A, EC 3.2.1.1.) with a detection limit of 0.2439 U/L—2200 U/L at 37°C and 405 nm of wavelength. The activity of protease was determined following instructions of Walter [23]. Casein 1% w/v was used as substrate in 0.2 M phosphate buffer at pH 7.0. One unit of protease indicates the amount of enzyme that releases 1 μg/ml/min of tyrosine determined at 660 nm of wavelength.

### 2.6. Plasma cortisol

The concentration of cortisol (ng/ml) in plasma was measured using a commercial ELISA kit (Calbiotech, USA, CAT No. CO368S, CID 5754) having a sensitivity of 1.16 ng/ml. The intra-assay and inter-assay precision were 3.8% and 8.65%, respectively (n = 24). The absorbance value was read on the ELISA reader at 450 nm. All the steps of the assay procedure were performed according to the manufacturer’s instructions.

### 2.7. Antioxidants assay

Plasma catalase (CAT) (U/ml) activity was determined using a commercial ELISA Colorimetric Activity Kit (Thermo Fisher Scientific, USA, CAT No. EIACATC, EC 1.11.1.6) having an analytical sensitivity of 0.052 U/ml. The absorbance was read at 560 nm at 37°C. The activity of superoxide dismutase (SOD) (ng/ml) were measured by using ELISA kit (PARS BIOCHME, China, CAT No. PRS-02005hu, EC 1.15.1.1) with an assay range of 0.3 ng/ml– 10 ng/ml. For malondialdehyde (MDA) (nmol/ml), ELISA kit (PARS BIOCHME, China, CAT No. PRS-00991hu, CAS 542-78-9) with an assay range of 0.3 ng/ml– 7 nmol/ml was used. Activity of glutathione peroxidase (GPx) (IU/ml) were measured by using ELISA kit (PARS BIOCHME, China, CAT No. PRS-00680hu, EC 1.11. 1.9) with an assay range of 3 IU/ml– 200 IU/ml. The absorbance value of SOD, MDA, and GPx was read at 450 nm and 37°C.

### 2.8. Statistical analysis

For all the statistical analysis, SPSS v.29 software was used. Data were shown as Mean± SE for all the parameters. Levene test were performed to check the homogeneity of variance of data. The effect of stocking density and time (month) on different parameters was determined by repeated-measures ANOVA. Repeated measure ANOVA was used to determine whether there is a statistically significant effect of between subject factor (stocking density) on different parameters. It also indicated whether a within subject factor (months) has a statistically significant impact or not. Along with that, it also provided statistical two-way interaction between the two factors i.e within subject (months) and between subject (density) (time*stocking density). Samplings were repeated on the same stocking density group every month which also makes this statistical test the most appropriate for the analysis. To reject the null hypothesis, a 0.05 probability level was used which indicates significant (P < 0.05) or insignificant (P>0.05) effect of stocking density, months, and combined effect of month*density. Degree of freedom (df) mentioned along each parameter was calculated as the sample size minus the number of restrictions. In case of stocking density, df was 2 as there were three treatment groups. In the case of months, it was 5 as there were six months. For the combined effect of month*density, df was 10 (2×5).

## 3. Results

### 3.1. Water quality parameters

Water temperature, pH, dissolved oxygen, ammonia and nitrites in all three treatment groups during the grow out period varied within the range of 33.96±0.41°C to 20.61±0.26°C, 7.17±0.87 to 8.87±0.03, 3.13±0.32 mg/L to 10.85±1.41 mg/L, 0.78±0.02 ppm to 1.32±0.02 ppm and 0.10±0.01 mg/L to 0.21±0.01 mg/L, respectively throughout the study period (S1 Table in S1 File). The detailed monthly variations (Mean ± SE) in water temperature (°C), pH, dissolved oxygen (mg/L), ammonia (ppm) and nitrites (mg/L) in all density groups, measured at four different times in 24 h between June till November has been presented as the supporting data in the form of table (S1 Table in S1 File).

### 3.2. Growth

A marked elevation was discerned in the growth of fish across all the experimental cohorts, denoted by the progressive augmentation in both total body length and total body weight throughout the duration of the trial (Table 1). A statistically significant subdued growth rate was evident within the HD treatment as substantiated by its average daily per fish weight gain of 4.21 g, in contrast to the weight gains observed in the MD treatment at 4.82 g and LD treatment at 4.74 g (df_2_, F = 52.493, P = 0.000). Although growth rate is statistically significant between treatment, no large difference has been observed quantitatively. The average daily per fish weight gain was above 4 g in all three treatments. No difference in the survival rate was observed between the treatments as it was above 99% in all the treatments. FCR measured in LD, MD and HD treatment was also similar as it was 1.90, 1.89 and 1.88 respectively.

### 3.3. Condition Factor (K) (%), Specific Growth Rate (SGR) (%), and Hepatosomatic Index (HSI) (%)

**Fig 2:**
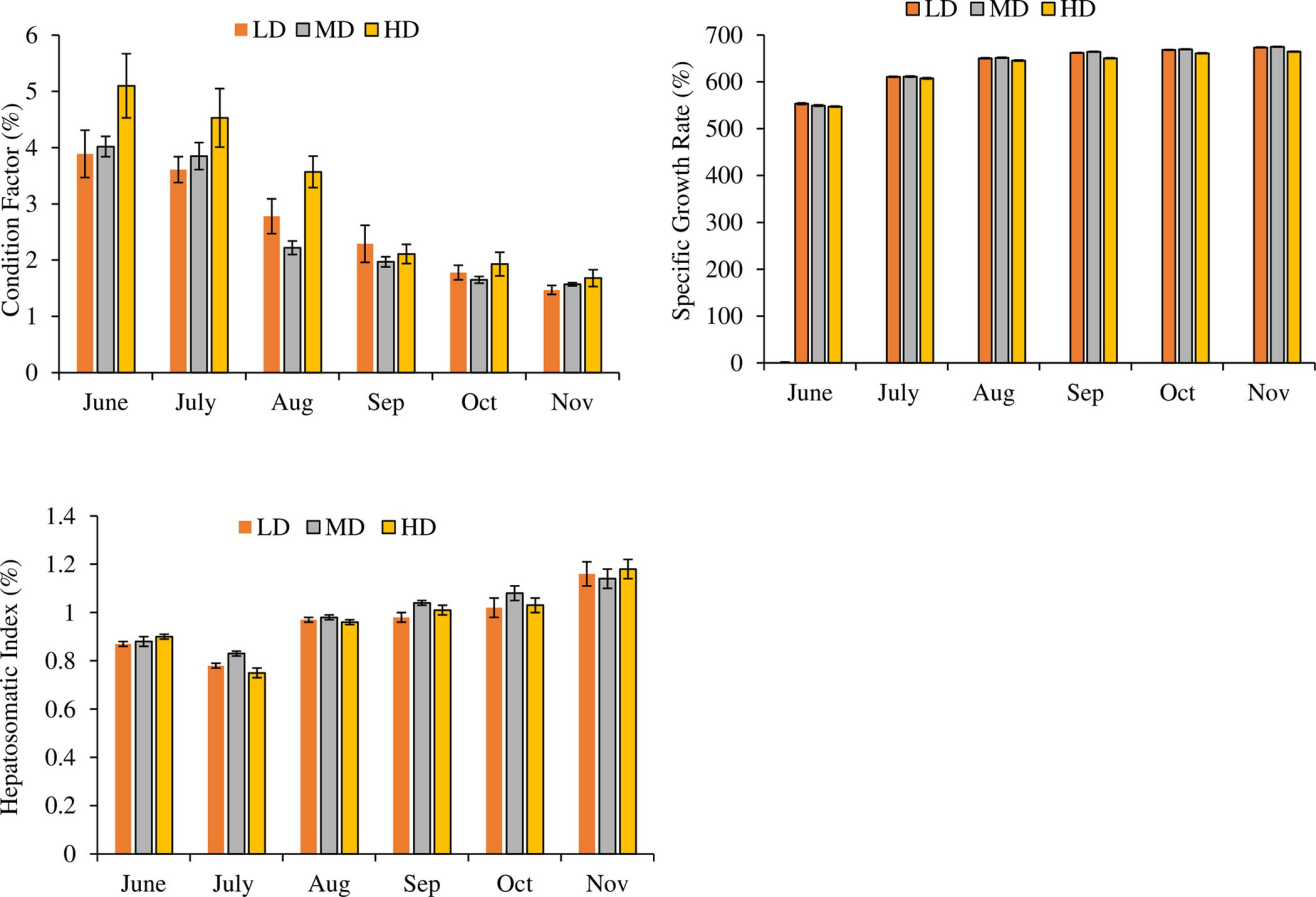
Monthly variations in the condition factor, specific growth rate and hepatosomatic index (Mean ± SE) determined in low density (LD), medium density (MD), high density (HD) groups over the period of six months.

Repeated measure ANOVA indicated that stocking density had a significant effect (P = 0.000) on condition factor (df_2_, F = 254.445), specific growth rate (df_2_, F = 238.768) and hepatosomatic index (P = 0.011; df_2_, F = 4.606) (Fig 2). Quantitative differences in the hepatosomatic index were not large between treatments despite being statistically different. Monthly variations in condition factor (df_5_, F = 37.584), specific growth rate (df_5_, F = 8223.703) and hepatosomatic index (df_5_, F = 215.220) were also significant (P = 0.000) between three treatments. In addition to this, month*density also had a significant effect (P = 0.000) on condition factor (df_10_, F = 15.774), specific growth rate (df_10_, F = 4.010), and hepatosomatic index (P = 0.010; df_10_, F = 2.348). SGR was recorded within the range of 553.36 ± 1.67 to 673.51 ± 0.68 in LD; 549.46 ± 1.43 to 674.87 ± 0.58 in MD and 547.32 ± 1.03 to 664.60 ± 0.75 in HD. A statistically significant decrease in the values of SGR and HSI was noted in the HD treatment as compared to LD and MD. The values of HSI were within the range of 0.75 ± 0.02 to 1.20 ± 0.04.

### 3.4. Proximate composition

**Fig 3:**
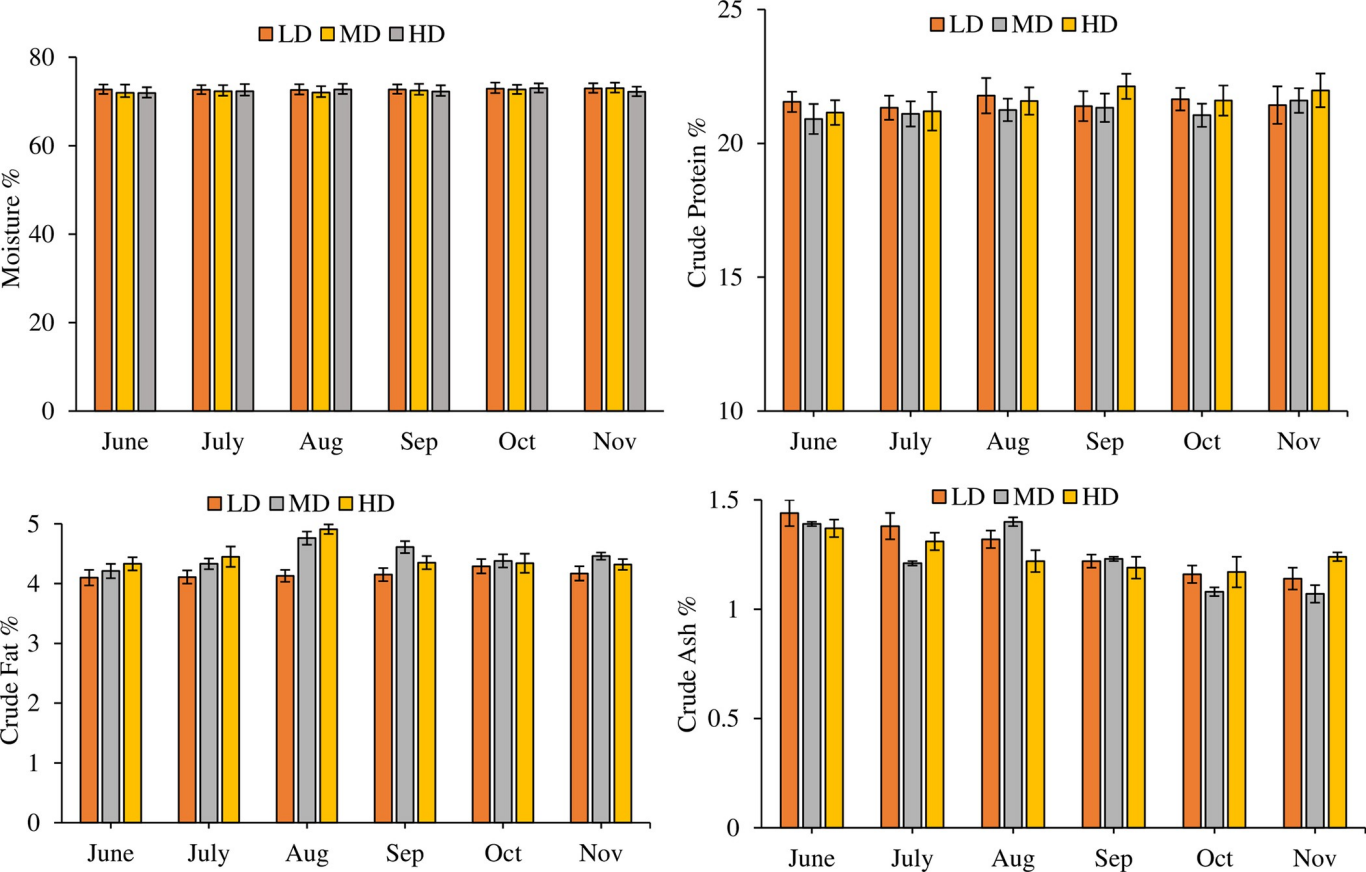
Monthly variations (Mean±SE) in proximate composition (%) of muscle samples in low density (LD), medium density (MD), high density (HD) treatments over the period of six months.

An insignificant difference was found between the content of moisture (df_2_, F = 0.359, P = 0.699), crude protein (df_2_, F = 2.455, P = 0.089) and crude ash (df_2_, F = 3.026, P = 0.051) in all density treatments (Fig 3). However, content of crude fat was significantly different in density treatment groups (df_2_, F = 26.796, P = 0.000) but this difference was not quantitatively large. Monthly variations in the content of moisture (df_5_, F = 0.334, P = 0.892), crude protein (df_5_, F = 1.233, P = 0.291) was insignificant. However, in case of crude ash (df_5_, F = 34.257) and crude fat (df_5_, F = 10.134) effect of time (month) was significant (P = 0.000). Interactive effect of month*density on moisture (df_10_, F = 0.170, P = 0.998), crude protein (df_10_, F = 0.643, P = 0.778) was insignificant. While, in case of crude ash (df_10_, F = 4.972) and crude fat (df_10_, F = 4.560) combined effect of month*density was significant (P = 0.000). In all the treatments, the content of crude protein ranged between 20.91 ± 0.56% to 22.13 ± 0.47%; crude fat within the range of 4.10 ± 0.13% to 4.91 ± 0.08% and crude ash within the range of 1.07 ± 0.04% to 1.44 ± 0.06%.

### 3.5. Amino acids profile

Among essential amino acids, methionine (df_2_, F = 1.207, P = 0.302) and threonine (df_2_, F = 1.163, P = 0.315) were not significantly different between density treatment groups (Table 2). In rest of the essential amino acids, a significant (P = 0.000) difference was observed between density treatment groups. Monthly variations in all of the essential amino acids were also significant (P = 0.000). Among essential amino acids, leucine, lysine, and arginine were the highest, and ornithine level was the lowest in all treatments. In case of non-essential amino acids (NEAA), cysteine (df_2_, F = 0.542, P = 0.582), serine (df_2_, F = 1.377, P = 0.255), glutamic acid (df_2_, F = 0.273, P = 0.761) and proline (df_2_, F = 1.554, P = 0.214) were insignificantly different between LD, MD and HD treatments (Table 3). Remaining non-essential amino acids (aspartic acid, asparagine, glycine, alanine, tyrosine) differed significantly (P = 0.000) between density treatment groups. Monthly variations in NEAA were also significant (P = 0.000). Glutamic acid was the highest and the lowest was cysteine in all treatments among non-essential amino acids (NEAA). Among all the amino acids, whether essential or non-essential in which statistically significant differences were observed between the treatments, the differences were not large quantitatively that can be considered as practically significant.

**Table 2:** Monthly variations (Mean±SE) in essential amino acids (Mean ± SE) in muscle samples of LD, MD, HD treatments over the period of six months. Values are expressed as mg of amino acid per g of crude protein (mg/gcp).

	**Essential Amino Acids**					
	**Methionine**					
**Group**	**June**	**July**	**August**	**September**	**October**	**November**
**LD**	23.16±0.42	23.44±0.43	22.23±1.09	23.71±0.35	23.98±0.39	23.59±0.38
**MD**	22.82±0.60	23.42±0.48	23.72±0.46	24.05±0.38	24.74±0.48	22.38±0.42
**HD**	22.68±0.56	22.48±0.43	22.78±0.38	24.08±0.31	24.63±0.61	22.66±0.68
**Threonine**						
**Group**	**June**	**July**	**August**	**September**	**October**	**November**
**LD**	24.32±0.53	24.49±0.75	24.93±0.58	25.26±0.74	25.73±0.84	25.79±0.71
**MD**	24.48±0.83	24.86±0.64	25.38±0.66	23.91±0.68	25.26±0.62	26.38±0.65
**HD**	26.36±0.90	25.86±0.93	26.46±1.17	22.92±0.69	24.50±0.68	26.85±1.19
**Valine**						
**Group**	**June**	**July**	**August**	**September**	**October**	**November**
**LD**	42.14±0.92	42.44±0.91	42.91±0.90	43.12±1.02	42.91±0.77	42.61±0.87
**MD**	41.88±1.10	41.94±1.13	42.08±1.05	41.94±1.04	42.00±0.83	41.88±0.94
**HD**	39.99±0.83	43.58±0.70	43.67±0.66	44.20±0.58	42.75±1.08	42.51±1.01
**Isoleucine**						
**Group**	**June**	**July**	**August**	**September**	**October**	**November**
**LD**	36.22±0.81	36.59±0.64	36.81±0.63	36.87±0.72	36.22±0.85	35.88±0.58
**MD**	34.98±1.02	35.02±0.94	35.05±0.66	35.37±0.49	33.21±1.11	34.63±0.61
**HD**	34.66±0.82	34.55±0.64	34.46±0.95	35.99±0.66	32.00±1.20	33.20±0.97
**Leucine**						
**Group**	**June**	**July**	**August**	**September**	**October**	**November**
**LD**	62.44±0.86	62.51±0.78	62.62±1.03	62.69±0.84	62.13±0.85	61.93±1.38
**MD**	64.54±1.29	63.85±0.88	63.64±0.99	64.30±1.03	62.19±1.11	61.00±1.37
**HD**	64.87±1.40	64.66±1.35	64.81±1.48	64.30±1.36	62.25±1.27	61.90±0.56
**Phenylalanine**						
**Group**	**June**	**July**	**August**	**September**	**October**	**November**
**LD**	39.76±1.17	40.19±1.18	40.41±1.24	40.61±0.87	40.51±1.19	41.08±0.95
**MD**	38.17±0.90	38.68±0.75	38.97±0.93	40.72±0.91	41.03±0.77	41.25±00.57
**HD**	38.52±1.02	38.77±0.97	38.48±1.11	39.20±0.99	39.00±1.04	40.01±1.00
**Histidine**						
**Group**	**June**	**July**	**August**	**September**	**October**	**November**
**LD**	36.65±1.76	36.42±1.84	36.51±1.45	36.71±1.36	37.14±0.93	35.53±1.01
**MD**	37.84±1.59	37.05±1.58	37.95±1.59	36.71±1.27	37.33±1.18	34.25±1.38
**HD**	35.19±0.74	36.30±1.41	37.47±1.27	35.80±0.89	35.34±1.34	33.80±1.25
**Lysine**						
**Group**	**June**	**July**	**August**	**September**	**October**	**November**
**LD**	64.12±1.07	64.56±0.95	64.91±1.12	64.61±1.03	63.75±1.23	63.22±1.18
**MD**	64.07±0.54	63.93±0.39	64.10±0.51	66.09±0.57	62.82±0.93	62.38±0.89
**HD**	62.88±0.65	63.01±0.72	62.91±0.63	63.16±0.62	61.25±0.88	62.03±0.89
**Arginine**						
**Group**	**June**	**July**	**August**	**September**	**October**	**November**
**LD**	53.89±0.87	54.69±0.71	54.91±0.63	55.15±0.87	56.39±0.84	60.04±1.27
**MD**	52.91±0.64	53.90±0.54	51.41±0.53	53.29±0.55	53.97±1.11	62.38±1.12
**HD**	51.96±0.68	53.13±0.74	50.76±0.75	53.54±0.69	54.38±0.97	60.20±1.51
**Ornithine**						
**Group**	**June**	**July**	**August**	**September**	**October**	**November**
**LD**	2.40±0.11	2.18±0.07	2.46±0.10	2.33±0.07	2.10±0.11	1.94±0.11
**MD**	2.56±0.07	2.32±0.08	2.44±0.09	2.41±0.08	2.17±0.06	1.88±0.07
**HD**	2.51±0.08	2.28±0.08	2.41±0.06	2.53±0.07	2.15±0.11	2.15±0.14

**Table 3:** Monthly variations (Mean±SE) in non-essential amino acids (Mean ± SE) in muscle samples of LD, MD, HD treatments over the period of six months. Values are expressed as mg of amino acid per g of crude protein (mg/gcp).

**Non-essential Amino Acids**						
**Cysteine**						
**Group**	**June**	**July**	**August**	**September**	**October**	**November**
**LD**	8.41±0.19	8.36±0.18	8.39±0.15	8.19±0.17	7.96±0.18	9.68±0.06
**MD**	8.45±0.09	8.39±0.10	8.35±0.08	8.23±0.11	8.00±0.15	9.86±0.05
**HD**	8.39±0.13	8.42±0.13	8.46±0.12	8.35±0.14	8.08±0.15	9.45±0.14
**Aspartic Acid**						
**Group**	**June**	**July**	**August**	**September**	**October**	**November**
**LD**	67.21±0.89	72.22±0.97	67.24±0.94	67.09±0.87	63.98±0.73	61.15±0.53
**MD**	68.89±0.70	73.11±0.71	73.04±0.92	68.36±0.63	64.71±0.52	63.16±0.64
**HD**	69.36±0.73	73.79±0.61	73.97±0.88	67.57±1.00	63.82±0.94	62.38±1.07
**Asparagine**						
**Group**	**June**	**July**	**August**	**September**	**October**	**November**
**LD**	65.06±1.03	73.21±0.97	65.27±0.49	70.13±1.32	63.99±0.73	61.21±0.52
**MD**	66.74±0.59	74.41±1.09	74.30±0.52	71.53±1.11	62.43±0.77	60.63±0.48
**HD**	68.20±1.39	74.02±0.61	73.97±0.63	70.20±1.26	62.64±1.73	59.36±0.86
**Serine**						
**Group**	**June**	**July**	**August**	**September**	**October**	**November**
**LD**	35.63±0.74	36.82±0.63	35.81±0.59	37.93±0.68	35.12±0.49	35.88±0.50
**MD**	36.91±0.85	37.79±0.84	37.85±0.81	37.85±0.79	34.75±0.72	36.58±0.69
**HD**	37.53±0.58	38.29±0.98	38.33±0.83	36.71±0.93	33.72±1.08	36.00±0.88
**Glutamic Acid**						
**Group**	**June**	**July**	**August**	**September**	**October**	**November**
**LD**	108.56±1.14	108.67±1.12	107.78±1.05	109.34±1.26	110.51±1.46	112.44±1.58
**MD**	106.67±0.98	107.26±0.95	105.57±0.76	112.91±1.69	109.38±1.60	113.80±1.20
**HD**	107.15±0.91	107.32±1.47	103.85±0.99	112.63±1.44	112.18±1.68	112.38±1.57
**Glycine**						
**Group**	**June**	**July**	**August**	**September**	**October**	**November**
**LD**	60.59±1.04	61.18±0.99	59.53±1.14	60.11±1.01	59.77±1.11	60.08±1.01
**MD**	62.21±1.01	62.18±1.04	62.03±1.04	60.25±0.73	58.40±1.05	63.16±0.73
**HD**	62.76±0.93	62.87±1.10	62.82±1.11	61.90±1.09	57.49±1.58	62.38±1.29
**Alanine**						
**Group**	**June**	**July**	**August**	**September**	**October**	**November**
**LD**	56.34±0.85	56.48±0.83	56.69±0.95	55.85±0.53	56.44±1.04	56.86±1.17
**MD**	55.34±0.90	55.92±0.62	53.16±0.73	56.01±0.80	55.50±0.92	57.22±0.80
**HD**	54.81±0.77	55.38±0.72	53.85±0.68	56.16±0.72	55.38±0.68	56.50±0.88
**Proline**						
**Group**	**June**	**July**	**August**	**September**	**October**	**November**
**LD**	38.63±0.94	38.71±0.95	37.94±0.89	39.44±0.60	39.98±0.73	40.12±0.52
**MD**	37.91±0.73	38.26±0.74	37.85±0.61	42.15±0.70	41.20±0.50 ^c^	38.61±0.51
**HD**	38.29±1.04	38.38±1.00	38.33±0.88	39.24±0.79	40.67±0.84	37.75±0.93
**Tyrosine**						
**Group**	**June**	**July**	**August**	**September**	**October**	**November**
**LD**	18.66±0.59	18.71±0.42	17.93±0.68	18.85±0.50	18.96±0.52	18.56±0.57
**MD**	18.11±0.71	17.99±0.43	17.91±0.69	18.99±0.49	18.63±0.58	17.44±0.61
**HD**	19.64±0.48	19.58±0.31	19.46±0.65	18.00±0.70	17.90±0.63	18.88±0.79

### 3.6. Fatty acids profile

Statistical analysis of saturated fatty acids (SFA) determined from muscle samples indicated that arachidic Acid was insignificantly different (df_2_, F = 0.224, P = 0.800) between the three density treatments (Table 4). Whereas, rest of the SFA differed significantly (P = 0.000). The monthly variations in all of the treatments were also significant (P = 0.000). In case of unsaturated fatty acids (UFA), palmitoleic acid (df_2_, F = 0.439, P = 0.645), oleic acid (df_2_, F = 0.403, P = 0.669) and decosapentanoic acid (df_2_, F = 1.655, P = 0.194) were insignificantly different between LD, MD and HD treatments (Tables 5 and 6). Rest of the UFA differed significantly (P = 0.000) between density treatments. Along with that, monthly variations in UFA were also significant (P < 0.05). The differences in the content of fatty acids (saturated and unsaturated) were not quantitatively large that it can be considered as practically significant difference although it was significantly different statistically.

**Table 4:** Monthly variations in saturated fatty acids (Mean ± SE) in total lipids extracted from muscle samples of LD, MD, HD treatments over the period of six months. Values are expressed as percentages of total fatty acids.

**C14:0—Myristic acid**						
**Group**	**June**	**July**	**August**	**September**	**October**	**November**
**LD**	0.22±0.01	0.23±0.01	0.26±0.01	0.30±0.01	0.30±0.01	0.34±0.01
**MD**	0.23±0.01	0.24±0.01	0.32±0.01	0.32±0.01	0.34±0.01	0.32±0.01
**HD**	0.23±0.01	0.23±0.01	0.30±0.01	0.31±0.01	0.34±0.01	0.36±0.01
**C15:0—Pentadecyclic Acid**						
**Group**	**June**	**July**	**August**	**September**	**October**	**November**
**LD**	0.21±0.01	0.20±0.01	0.20±0.01	0.23±0.01	0.25±0.01	0.24±0.01
**MD**	0.22±0.01	0.22±0.01	0.24±0.01	0.21±0.01	0.23±0.01	0.24±0.01
**HD**	0.20±0.01	0.21±0.01	0.22±0.01	0.22±0.01	0.24±0.01	0.23±0.01
**C16:0—Palmitic Acid**						
**Group**	**June**	**July**	**August**	**September**	**October**	**November**
**LD**	30.25±0.57	30.28±0.64	30.18±0.57	29.73±0.58	29.55±0.57	29.03±0.65
**MD**	31.08±0.62	31.58±0.64	32.43±0.59	30.20±0.61	31.43±0.54	29.30±0.64
**HD**	31.90±0.56	31.83±0.74	32.40±0.73	30.03±0.60	31.40±0.85	29.30±0.63
**C18:0—Stearic Acid**						
**Group**	**June**	**July**	**August**	**September**	**October**	**November**
**LD**	5.65±0.15	5.69±0.14	5.70±0.11	5.63±0.13	5.62±0.10	5.72±0.10
**MD**	5.48±0.16	4.39±0.14	5.51±0.13	5.58±0.12	5.59±0.07	5.41±0.11
**HD**	5.23±0.11	5.35±0.09	5.50±0.09	5.54±0.12	5.59±0.10	5.40±0.13
**C20:0—Arachidic Acid**						
**Group**	**June**	**July**	**August**	**September**	**October**	**Novembe**
**LD**	2.77±0.09	2.83±0.08	2.91±0.07	2.97±0.10	2.81±0.10	2.74±0.09
**MD**	2.83±0.08	2.89±0.07	2.91±0.09	3.02±0.10	2.71±0.08	2.74±0.07
**HD**	2.85±0.08	2.94±0.07	2.90±0.08	3.00±0.09	2.78±0.08	2.65±0.09

**Table 5:** Monthly variations in monounsaturated fatty acids (Mean ± SE) in total lipids extracted from muscle samples of LD, MD, HD treatments over the period of six months. Values are expressed as percentages of total fatty acids.

**C14:1 n-5—Tetrasenoic Acid**						
**Group**	**June**	**July**	**August**	**September**	**October**	**November**
**LD**	0.67±0.03	0.65±0.02	0.59±0.03	0.85±0.02	0.87±0.02	0.49±0.05
**MD**	0.70±0.04	0.65±0.03	0.40±0.04	0.87±0.03	0.90±0.03	0.52±0.04
**HD**	0.72±0.04	0.70±0.02	0.91±0.06	0.89±0.04	0.90±0.02	0.52±0.05
**C15:1 n-5—Pentadecenoic Acid**						
**Group**	**June**	**July**	**August**	**September**	**October**	**November**
**LD**	0.29±0.02	0.31±0.02	0.34±0.02	0.28±0.01	0.33±0.02	0.32±0.02
**MD**	0.30±0.02	0.33±0.02	0.31±0.01	0.34±0.02	0.35±0.02	0.32±0.02
**HD**	0.33±0.02	0.34±0.01	0.32±0.02	0.31±0.01	0.34±0.02	0.37±0.02
**C16:1 n-7—Palmitoleic Acid**						
**Group**	**June**	**July**	**August**	**September**	**October**	**November**
**LD**	4.87±0.06	4.94±0.16	4.86±0.05	4.34±0.05	4.31±0.07	4.91±0.06
**MD**	4.79±0.07	4.87±0.17	4.80±0.05	4.37±0.06	4.28±0.08	4.97±0.06
**HD**	4.81±0.05	4.90±0.14	4.82±0.04	4.42±0.07	4.27±0.08	5.03±0.11
**C18:1 n-9—Oleic Acid**						
**Group**	**June**	**July**	**August**	**September**	**October**	**November**
**LD**	33.53±0.66	33.55±0.67	33.61±0.54	33.71±0.47	33.68±0.77	33.70±0.69
**MD**	33.28±0.64	33.33±0.66	33.33±0.67	34.50±0.47	32.50±0.78	33.70±0.68
**HD**	33.38±0.70	33.49±0.66	33.35±0.54	34.52±0.55	32.53±0.73	33.71±0.70
**Total Monounsaturated Fatty Acids (MUFA)**						
**LD**	39.36±7.96	39.45±7.97	39.40±7.98	39.18±8.02	39.19±8.00	39.42±8.01
**MD**	39.07±7.90	39.18±7.91	38.84±7.94	40.08±8.20	38.03±7.71	39.51±8.01
**HD**	39.24±7.92	39.43±7.94	39.40±7.89	40.14±8.21	38.04±7.72	39.63±8.00

**Table 6:** Monthly variations in polyunsaturated fatty acids (Mean ± SE) in total lipids extracted from muscle samples of LD, MD, HD treatments over the period of six months. Values are expressed as percentages of total fatty acids.

**Group**	**June**	**July**	**August**	**September**	**October**	**November**
**C18:2 n-6—Linoleic Acid**						
**LD**	16.93±0.16	16.94±0.14	16.97±0.09	17.16±0.17	16.37±0.15	16.08±0.08
**MD**	17.05±0.17	17.31±0.15	17.34±0.09	17.33±0.18	16.56±0.14	16.00±0.10
**HD**	17.11±0.13	17.28±0.14	17.10±0.11	17.36±0.13	16.88±0.11	16.21±0.14
**C18:3 n-3—α-linolenic Acid**						
**Group**	**June**	**July**	**August**	**September**	**October**	**November**
**LD**	3.37±0.06	3.36±0.07	3.34±0.06	3.40±0.07	3.42±0.05	3.49±0.06
**MD**	3.40±0.06	3.50±0.08	3.78±0.07	3.35±0.06	3.49±0.06	3.37±0.06
**HD**	3.44±0.08	3.27±0.08	3.10±0.12	3.41±0.07	3.35±0.12	3.26±0.12
**C20:5 n-3—Eicosapentanoic Acid**						
**Group**	**June**	**July**	**Augus**	**September**	**October**	**November**
**LD**	4.31±0.09	4.36±0.05	4.39±0.09	5.17±0.09	5.38±0.10	5.39±0.12
**MD**	4.66±0.09	4.71±0.08	5.95±0.09	5.72±0.12	5.57±0.10	5.61±0.11
**HD**	4.49±0.18	4.64±0.21	4.20±0.10	5.44±0.27	5.62±0.22	5.42±0.26
**C22:5 n-3—Decosapentanoic Acid**						
**Group**	**June**	**July**	**August**	**September**	**October**	**November**
**LD**	1.48±0.07	1.50±0.05	1.49±0.06	1.52±0.04	1.51±0.04	1.53±0.03
**MD**	1.42±0.06	1.46±0.06	1.48±0.05	1.45±0.04	1.51±0.04	1.50±0.02
**HD**	1.37±0.08	1.42±0.05	1.45±0.06	1.51±0.04	1.51±0.04	1.60±0.05
**C22:6 n-3—Decosahexanoic Acid**						
**Group**	**June**	**July**	**August**	**September**	**October**	**November**
**LD**	4.59±0.12	4.61±0.13	4.60±0.11	4.58±0.12	4.66±0.09	4.89±0.13
**MD**	4.61±0.12	4.71±0.13	5.32±0.11	4.87±0.11	4.88±0.13	5.17±0.13
**HD**	4.89±0.08	4.69±0.10	5.01±0.09	4.96±0.11	4.81±0.22	5.01±0.13
**n-3/n-6**						
**LD**	0.81±0.03	0.82±0.01	0.81±0.02	0.85±0.04	0.91±0.04	0.95±0.03
**MD**	0.83±0.02	0.83±0.02	0.95±0.04	0.89±0.03	0.93±0.04	0.98±0.05
**HD**	0.83±0.01	0.81±0.01	0.80±0.03	0.88±0.02	0.91±0.03	0.94±0.04

### 3.7. Blood hematology and biochemistry

**Fig 4:**
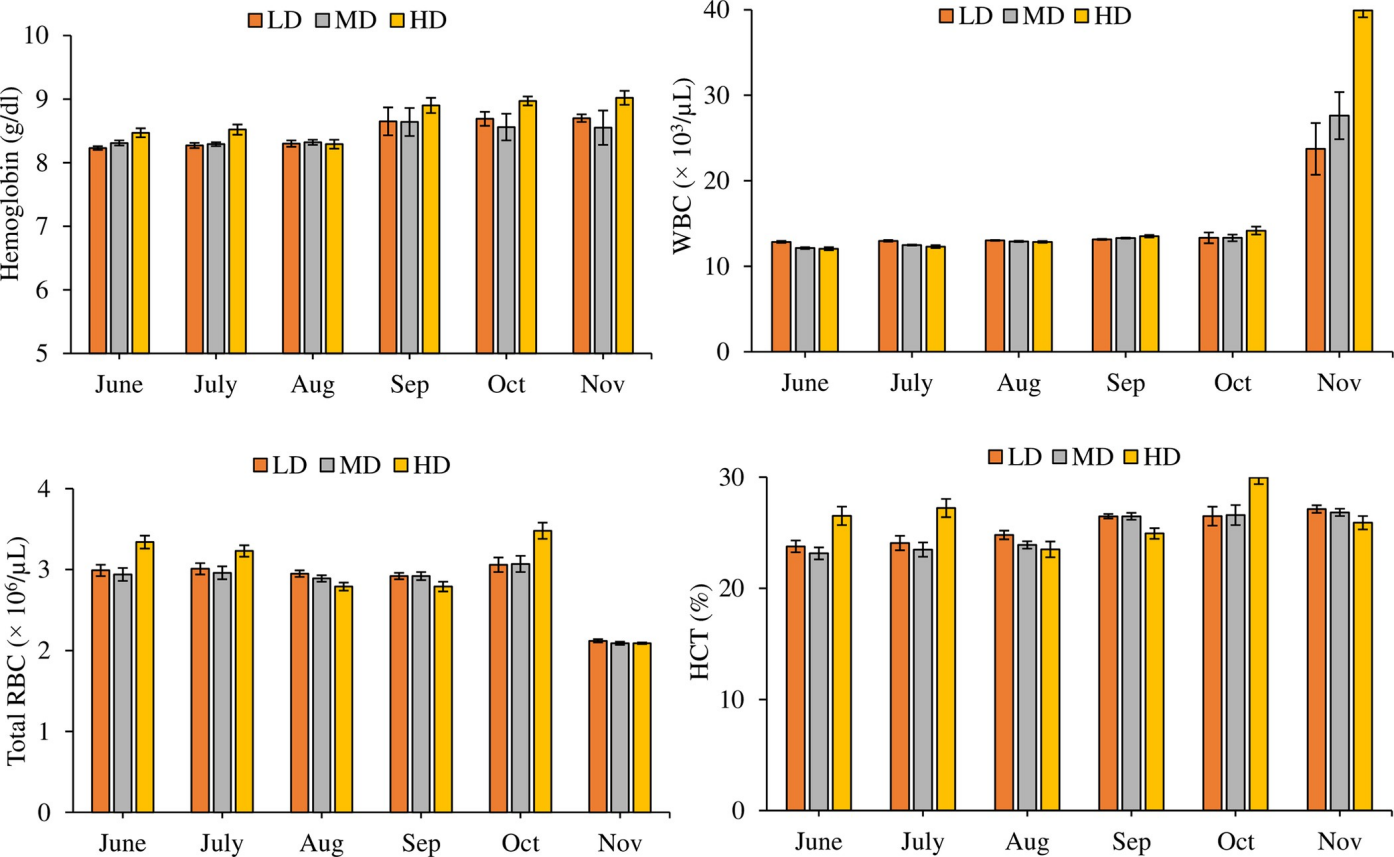
Monthly variations in blood hematological indices (Mean ± SE) (Hemoglobin, white blood cells (WBC), total red blood cells (RBC) and hematocrit (HCT)) from LD, MD, HD treatments over the period of six months.

**Fig 5:**
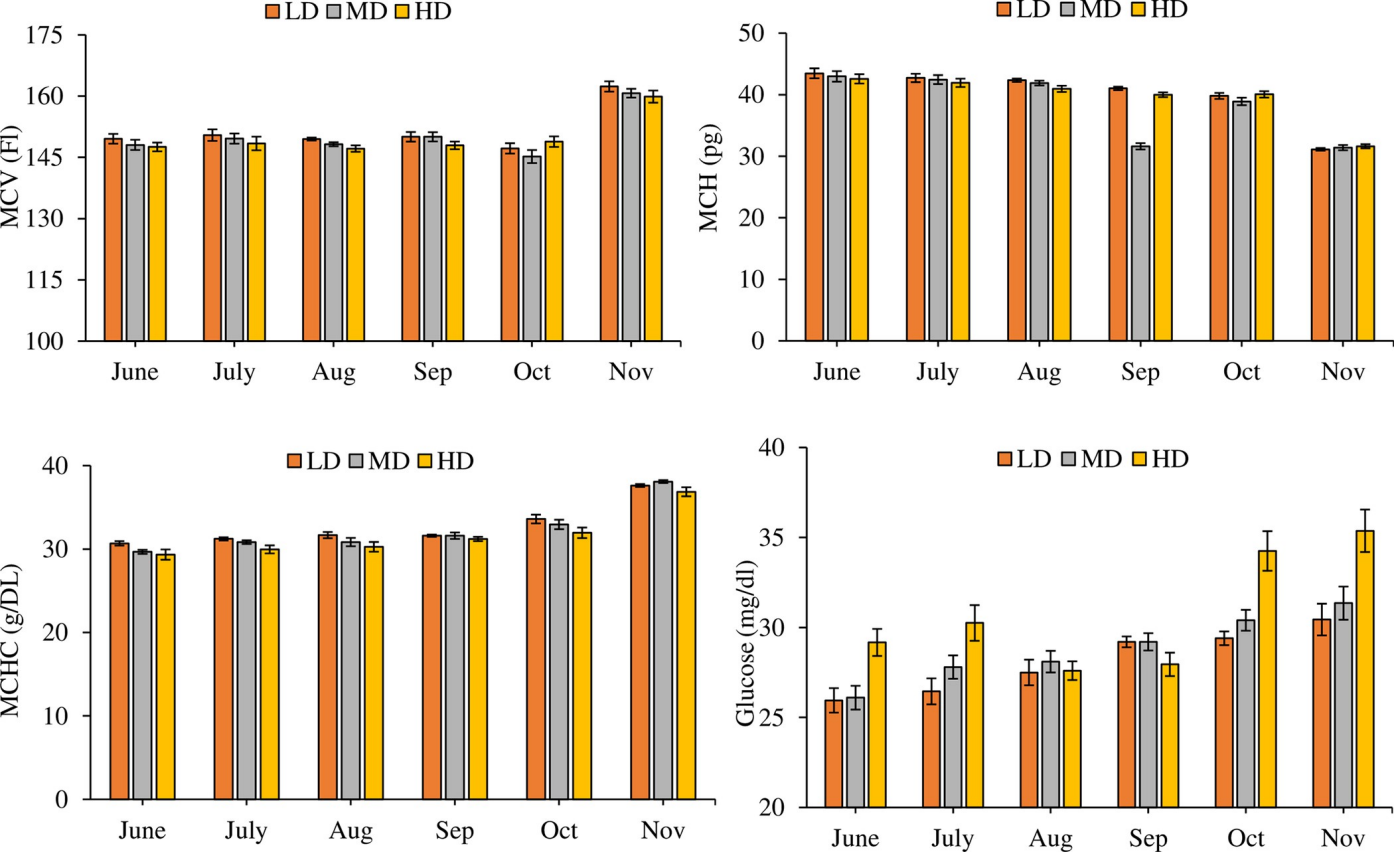
Monthly variations in blood hematological indices (Mean ± SE) (Mean corpuscular volume (MCV), Mean corpuscular hemoglobin (MCH) and mean corpuscular hemoglobin concentration (MCHC)) and glucose levels from LD, MD, HD treatments over the period of six months.

**Fig 6:**
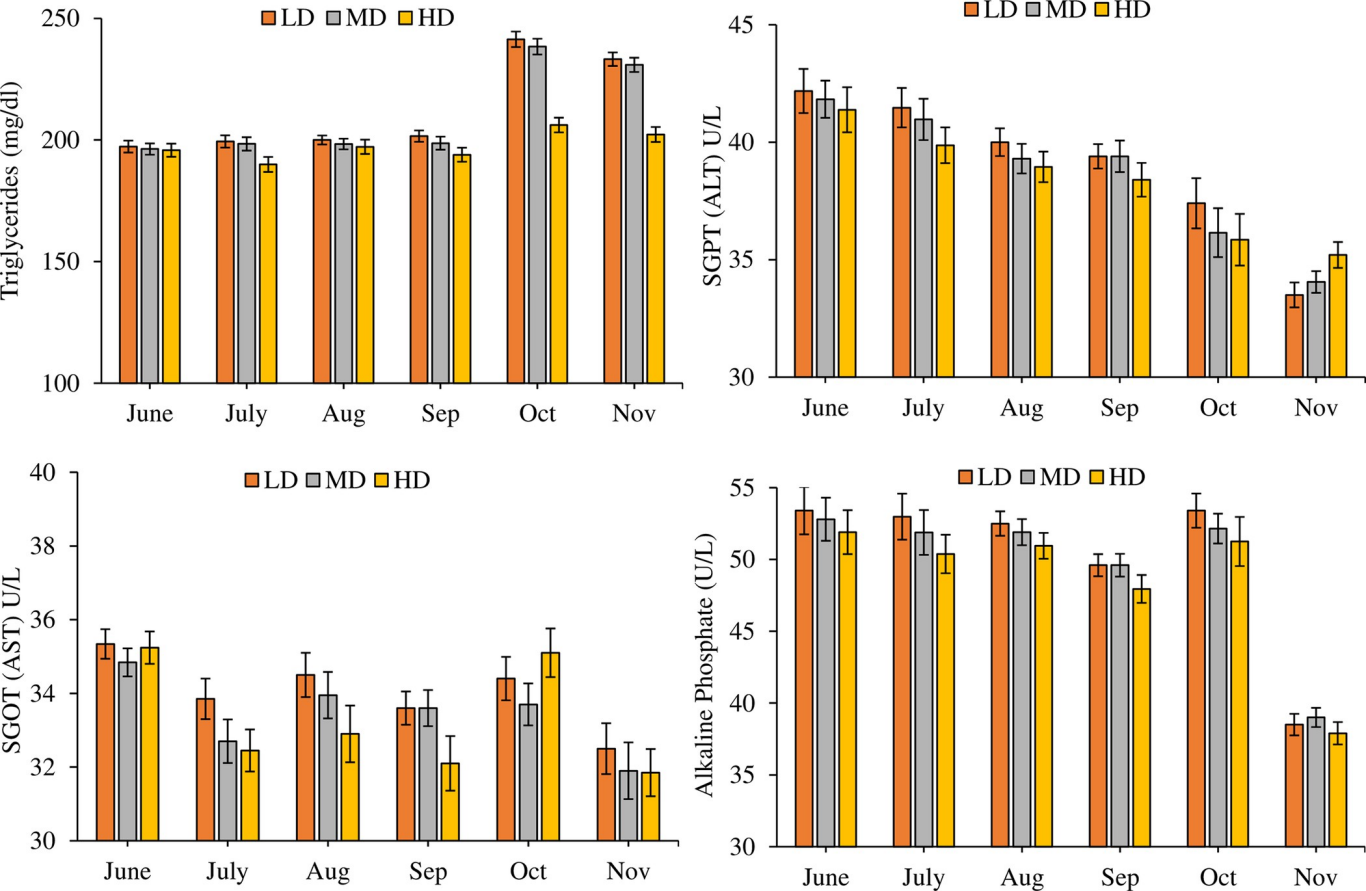
Monthly variations in blood biochemical parameters (Mean ± SE) (Triglycerides, Alanine aminotransferase (ALT), aspartate aminotransferase (AST) and alkaline phosphate) in plasma samples of LD, MD, HD treatments over the period of six months.

A significant increase (P = 0.000) was observed in the content of Hb (df_2_, F = 68.784), WBC (df_2_, F = 36.505), RBC (df_2_, F = 17.221) and HCT (df_2_, F = 53.326) in the high-density treatment as compared to LD and MD (Fig 4). A significant effect of density on MCV (df_2_, F = 9.463, P = 0.010), MCH (df_2_, F = 3.245, P = 0.039), MCHC (df_2_, F = 29.558, P = 0.000), GLU (df_2_, F = 68.817) AST (df_2_, F = 7.120), and ALT (df_2_, F = 6.140, P = 0.003) was observed statistically (Figs 5 and 6) but practically the differences were not so large. TG decreased significantly (df_2_, F = 62.322, P = 0.000) in HD towards the end of the trial (Fig 6). Monthly variations in all the hematological and biochemical parameters in the study were also significant (df_5_, P = 0.000). Besides independent effects of density and time on hematological and biochemical parameters, the interactive effect of month*density on most of these parameters were also significant (P < 0.05).

### 3.8. Digestive enzymes activity

**Fig 7:**
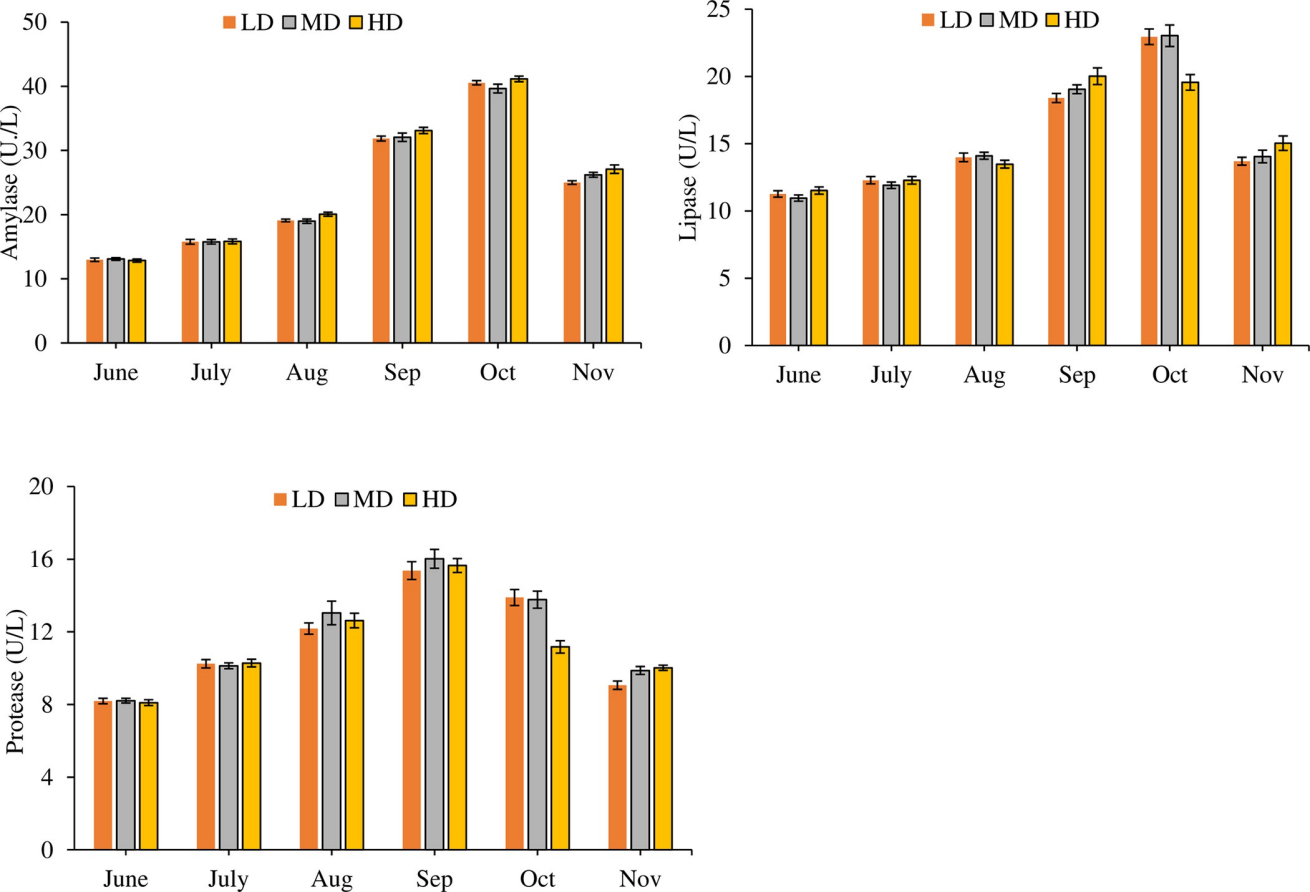
Monthly variations in in the activity of digestive enzymes (Mean ± SE) determined from viscera samples of low density (LD), medium density (MD), high density (HD) treatments over the period of six months.

Repeated measure ANOVA found that stocking density significantly affected the activity of amylase (df_2_, F = 27.636) and protease (df_2_, F = 11.703) (P = 0.000) but practically the difference cannot be considered quantitatively significant (Fig 7). Activity of lipase was insignificantly different in all the treatments (df_2_, F = 1.896, P = 0.153). Significant (P = 0.000) monthly variations in the activity of digestive enzymes were also observed in the study. In addition to this, combined effect of month*density on amylase (df_10_, F = 5.365), lipase (df_10_, F = 20.884) and protease (df_10_, F = 15.280) was also significant (P = 0.000). The increase in the enzymatic activity of amylase, lipase, and protease was evident up to the month of September during the trial. However, a subsequent decline was observed in the final two months (October and November) in all the treatments. The concentration of amylase, lipase, and protease in all density treatments of the study was within the range of 12.84±0.24 to 41.15±0.44, 10.96±0.23 to 23.03±0.80 and 8.10±0.16 to 16.02±0.52 respectively.

### 3.9. Profile of cortisol

**Fig 8:**
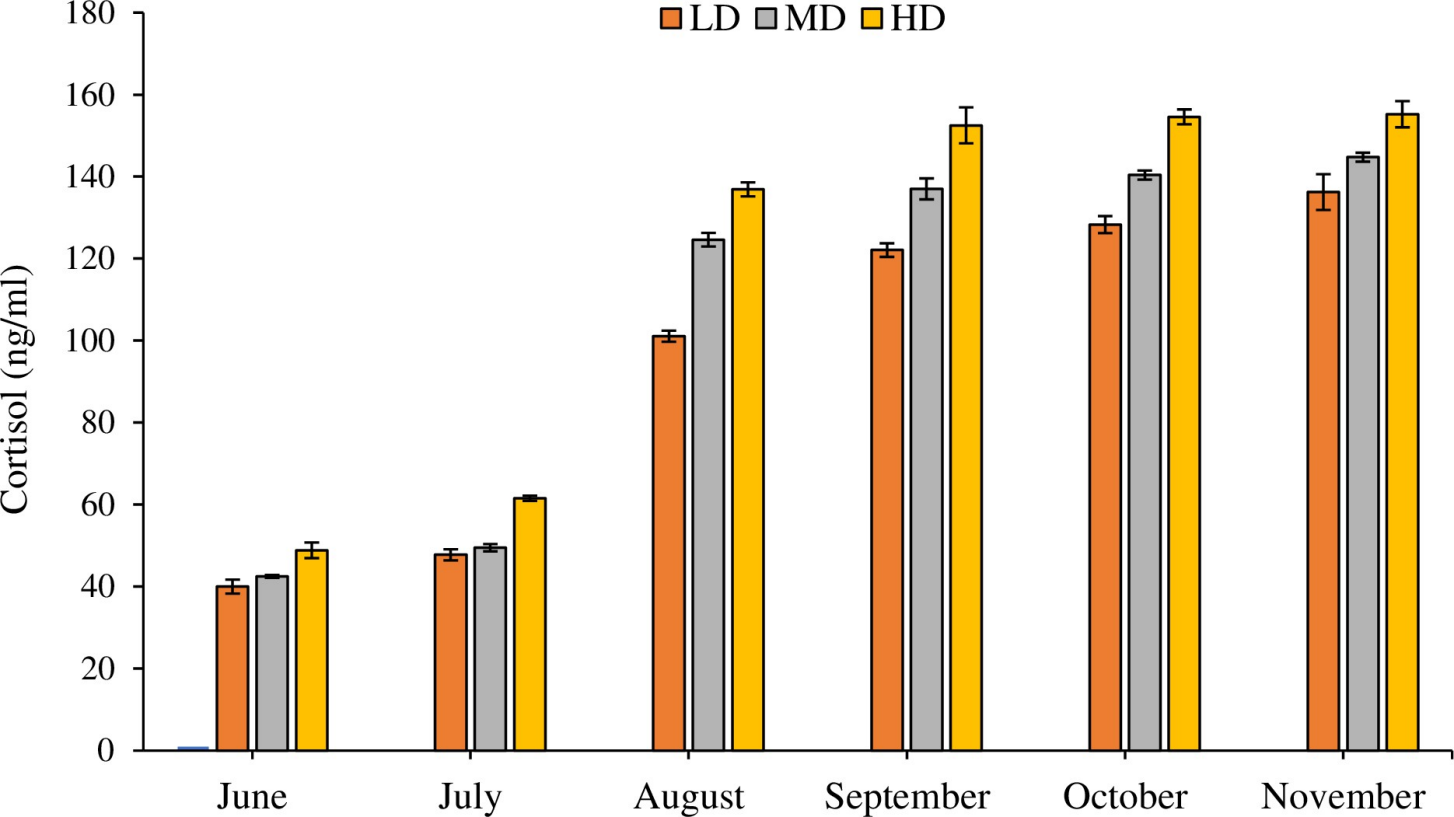
Monthly variations in the concentration of cortisol (Mean ± SE) determined in plasma samples of low density (LD), medium density (MD), high density (HD) groups over the period of six months.

The effect of stocking density on the concentration of cortisol was significant (df_2_, F = 6.299, P = 0.002) (Fig 8). Monthly variations in the concentration of cortisol were also significant (df_5_, F = 1095.791, P = 0.000). In addition to this, combined effect of month*density was insignificant (df_10_, F = 0.378, P = 0.956). There was no significant increase in concentration of cortisol during the initial months (June and July) of the trial. However, its level increased afterward and remained high till end of the study. Levels of cortisol in LD, MD, and HD treatment were within the range of 40.00±1.71 to 136.23±4.35 ng/ml, 42.49±0.34 to 144.74±3.11 ng/ml and 48.85±1.90 to 155.23±3.19 ng/ml respectively.

### 3.10. Antioxidants assay

**Fig 9:**
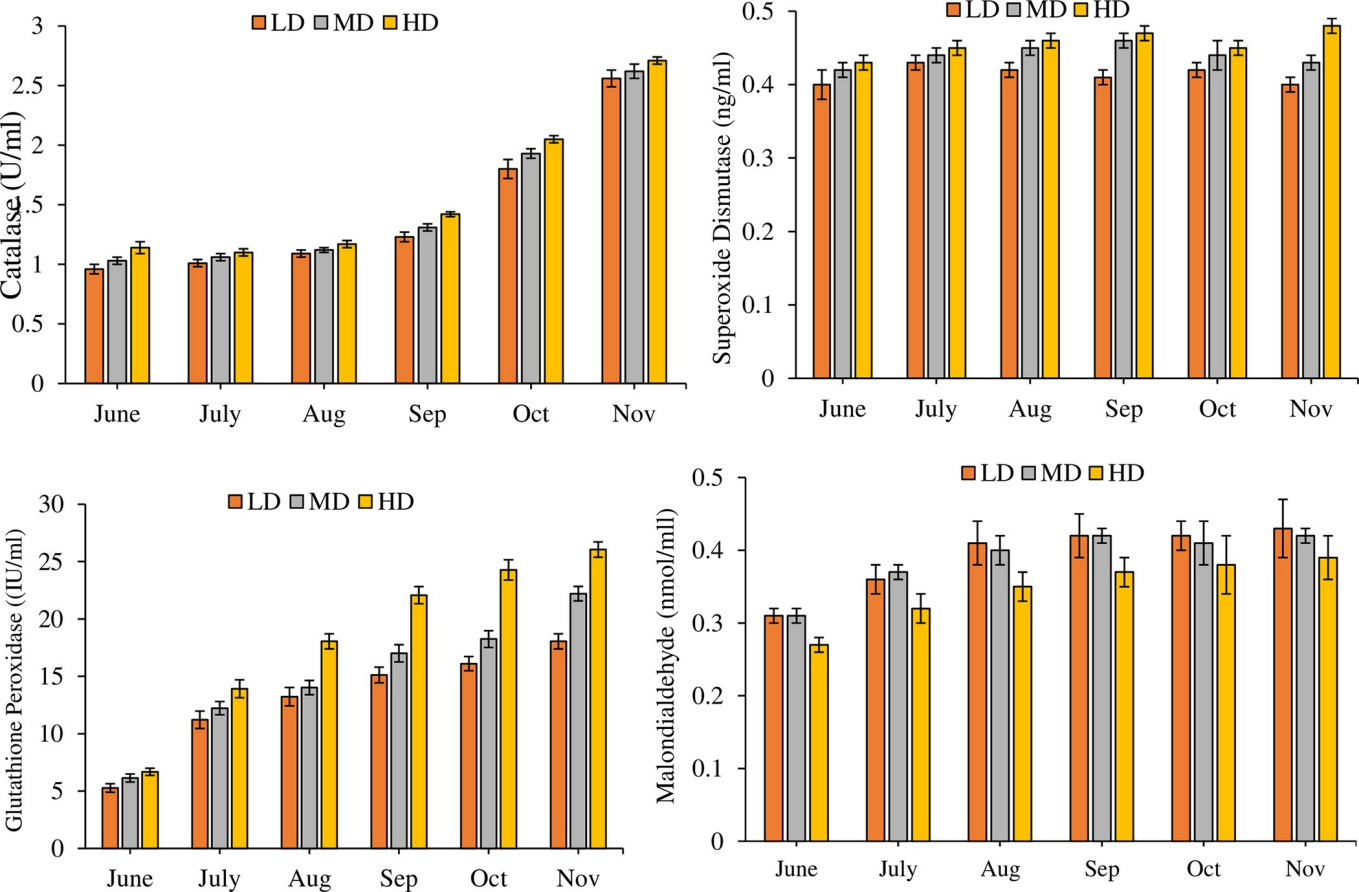
Monthly variations in the level of antioxidants (Mean ± SE) determined from blood plasma samples of LD, MD, HD treatments over the period of six months.

A significant difference (P = 0.000) was observed in the levels of CAT (df_2_, F = 47.110), SOD (df_2_, F = 21.129), MDA (df_2_, F = 25.127) and GPx (df_2_, F = 205.399) between all three density treatments (Fig 9). Monthly variations in the levels of CAT (df_5_, F = 1101.255), SOD (df_5_, F = 14.318), MDA (df_5_, F = 39.493) and GPx (df_5_, F = 678.437) in all the density treatment groups were significant (P = 0.0000). Impact of month*density on the levels of CAT (df_10_, F = 0.988, P = 0.452), SOD (df_10_, F = 1.305, P = 0.223), MDA (df_10_, F = 0.243, P = 0.992) was insignificant contrary to GPx where it showed a significant impact (df_10_, F = 15.762, P = 0.000).). Collectively, in all the treatments, concentration of CAT, SOD, MDA, and GPx was within the range of 0.96±0.04 U/ml to 2.71±0.03 U/ml, 0.40±0.01 ng/ml to 0.48±0.01 ng/ml, 0.27±0.01 nmol/ml to 0.43±0.04 nmol/ml and 5.27±0.37 IU/ml to 26.05±0.67IU/ml, respectively.

## 4. Discussion

The utilization of IPRS represents an innovative approach yielding optimal productivity and economic returns for aqua culturists, allowing a mere 2%– 5% of pond area for fish cultivation, allocating a substantial 95%–98% for efficient water purification purposes. However, various challenges can be linked to intensive fish rearing encompassing stress, disease prevalence, growth dynamics, and welfare of fish. Comparison between the outcomes of this investigation and prior IPRS data is tough, due to different circumstances such as IPRS design, climatic conditions, stocking area, stocking densities, study period, species, and overall pond area, number of raceways, initial stocking weight and average final size of fish. In this study, total harvested biomass was highest in HD (average 66.76 kg/m^3^) treatment as compared to MD (average 49.05 kg/m^3^) and LD (average 29.15 kg/m^3^) treatment. Similar harvested biomass was also reported in earlier studies in raceways [24] i.e 21,840 kg (5,288 kg/ha) for bluntnose black bream (*Acanthopagrus butcheri***)**, 17,850 kg (4,322 kg/ha) for channel catfish (*Ictalurus punctatus*), 12,960 kg (3,138 kg/ha) for yellow catfish (*Pylodictis olivaris*) and 10,750 kg (2,603 kg/ha) for largemouth bass (*Micropterus salmoides*) and ictalurid catfish 49,913 kg (20,540 kg/ha) [25].

The survival rate in all treatments was above 99% in this study was greater than previous studies reported for largemouth bass (86%), yellow catfish (90%), channel catfish (85%), bluntnose black bream (91%; [7] and hybrid catfish (89.1%; [25]). Amongst the array of parameters subjected to scrutiny for depicting stress, fish growth stands as a salient focus. In this contemporary investigation, the fish cohorts allocated as LD and MD exhibited enhanced acclimation within the confines of their respective rearing environments. Pertinently, no statistically discernible impact emerged across final body weight, WG, SGR, K, or HSI among the LD and MD treatments. These findings parallel the study on largemouth bass [7] and tilapia [26] which demonstrated resilience to elevated stocking densities. However, the HD treatment within the present study demonstrated statistically different growth. Analogously, an inverse correlation between stocking densities and the growth performance of Nile Tilapia (*Oreochromis niloticus*) was observed [27–29]. The factors contributing to reduced growth at high density potentially encompass reduced feed consumption, adversarial social interactions, and curtailed DO levels [9]. Statistically significant results such as growth in HD treatment was not quantitatively large to be practically meaningful or relevant. Statistical significance provides evidence of an effect or relationship, it doesn’t automatically imply that the observed effect is large enough to be practically important or useful in a real-world context. Therefore, statistical significance alone does not guarantee practical significance.

In the context of the present inquiry, the FCR within the LD, MD, and HD treatment demonstrated a notable synchrony, recording values of 1.90, 1.89, and 1.88, respectively, an outcome attributable to the exemplary husbandry parameters sustained throughout the study period. Antecedent literature has charted a spectrum of FCR figures in the realm of IPRS encompassing Bluntnose Black Bream (1.50), Yellow Catfish (1.51), and Largemouth Bass (1.22; [7], as well as range spanning Catfish (1.74–1.95; [25]) and Channel Catfish (1.45–1.57; [24]). Simultaneously, another study on tilapia stated unaltered FCR profiles, despite high stocking densities, the rationale attributed to the uniform dietary provisioning in similar conditions [30]. Typically, FCR below 2.0 is achieved in controlled pond such as in raceways where feeding is conducted with greater restraint which is better as compared to excessive feeding, higher FCR (2.2 to 3.0) but poor survival rate. A key factor of lower FCR (below 2.0) in raceway-type systems is the uniformity of fish size stocked mitigating the occurrence of cannibalism and competitive dominance of larger fish over their smaller counterparts [25].

The evaluation of muscular tissue nutritional composition, ascertained via amino and fatty acid profiles, remained impervious to the influence of elevated stocking density within this investigation. In case of amino acid composition, some amino acids were statistically different in density treatment groups while some were not, but all of the amino acids did not differ quantitatively in LD, MD and HD groups. These results are congruent with antecedent findings in the context of Grass Carp (*Ctenopharyngodon idellus*), wherein unaltered Total Amino Acid (TAA) and Total Essential Amino Acid (TEAA) levels were documented under high stocking density. Elevated levels of EAA may conceivably satiate augmented energy requisites and catalyse functional protein biosynthesis, serving as a response to the provocation of crowding-induced stress. Furthermore, the heightened content of NEAA in this examination refers to gluconeogenesis as an adaptive mechanism to elevate energy availability [31].

Considering the lipid composition, the fish examined in the present investigation exhibited a fat content ranging from 5% to 10% by mass, thereby classifying it as a fish of intermediate lipid content, in congruence with the findings of [32]. Within the context of freshwater species, the n-3/n-6 ratio adhered to the stipulated range of 1.6–2.0, in accordance with the guidelines [33]. This analogous outcome was documented in the case of largemouth bass cultivated under IPRS conditions, although with relatively diminished proportions of eicosapentaenoic acid (EPA) and docosahexaenoic acid (DHA) [34]. An augmentation in the levels of SFA and PUFA within the muscular tissue of grass carp was noted under conditions of high stocking density [31]. The nutritional profile of the cultivated fish in the current study demonstrated alignment with the dietary benchmarks, as evidenced by the prevalence of UFAs relative to SFAs, and the maintenance of a satisfactory range of total PUFA content, (n-6 and n-3 fatty acids) [35].

The enzymatic activities of digestion, encompassing amylase, lipase, and protease, exhibited a modest decrement towards the end of the experimental investigation. Similarly, diminished enzymatic functionalities of trypsin, amylase, and lipase manifested within the tilapia [36]. This phenomenon may be attributed to the potential disruption of the endocrine system causing a suppression in the activity of digestive enzymes and a concurrent elevation in cortisol levels during stress-inducing conditions [37]. Conditions of elevated stocking densities can potentially necessitate augmented energy expenditure to procure sustenance and effectively manage inter-species interactions amidst stress-inducing stimuli [7].

Conventionally, hematology and biochemical indices serve as pivotal markers of the comprehensive health status of fish. This investigation has unveiled an augmentation in hemoglobin (Hb) concentration within the HD cohort, juxtaposed with the LD and MD treatments. Analogous elevations in Hb concentration have been documented in juvenile Amur sturgeon [8] and tilapia [38] under conditions of crowding stress. In congruence with these findings, the current study demonstrates a substantial escalation in RBC count and HCT level within the HD treatment, mirroring congruent results in other studies involving tilapia [38] and amur sturgeon (*Acipenser schrenckii*) [8] exposed to heightened stocking densities. These discernible elevation in Hb concentration and RBC count are hypothesized to represent an adaptive response to heightened oxygen requisites, thereby facilitating the augmented carriage and conveyance of oxygen to body tissues [8]. Furthermore, the leukocyte (WBC) count also exhibited a surge within the HD treatment in this study, indicative of a robust immune competence analogous to the resilience observed in tilapia reared under IPRS conditions [38].

Elevated glucose concentrations were notably evident within the HD cohort in contrast to LD and MD treatments, demonstrating a coherent pattern with prior investigations. This trend of heightened glucose content has also been substantiated in tilapia [28], Senegalese sole (*Solea senegalensis*) [39], rainbow trout [9], and tilapia [38]. Elevated glucose levels are indicative of augmented energy requisites amidst crowding-induced stress conditions [8]. Concomitant with this inquiry, the plasma triglyceride (TG) levels demonstrated a decrement in the HD treatment towards the culmination of the experimentation, distinguishing them from the MD and LD treatments. Analogous findings are corroborated by parallel studies encompassing largemouth bass [7], senegalese sole [39], tilapia [28], amur sturgeon [8], and rainbow trout [9]. The diminishing TG levels indicate an escalation in energy consumption for stress adaptation, encompassing the metabolic conversion of triglycerides into glucose [9]. Furthermore, distinct alterations in the levels of AST, ALT, and ALP were not observed across the treatments within this study, substantiating the stability of liver function and the immune system. This corroborates analogous to another study where tilapia reared at various densities showcased undisturbed AST and ALT activity [40].

Cortisol, a pivotal biomarker of stress responsiveness, exhibited a noteworthy elevation in the HD treatment as compared to LD and MD treatments. This salient elevation in cortisol levels during stress exposure is also corroborated within the context of rainbow trout [41], senegalese sole [42], and channel catfish [43]. Intricately interlinked with the organism’s physiological dynamics, reactive oxygen species (ROS) exhibit a direct positive correlation with ambient oxygen concentrations. An organism can face oxidative stress if this ROS surpasses the organism’s capability to quench the damaging cellular components [44]. As a countermeasure against oxidative stress, an intricate array of antioxidants is inherently present, encompassing both enzymatic entities such as SOD, CAT, and GPx, and nonenzymatic molecules like ascorbic acid (vitamin C), glutathione, α-tocopherol (vitamin E), carotenoids, among others [45].

The plasma enzymatic activities of SOD, CAT, and GPx, as assessed within the scope of this investigation, evinced marked elevation in the HD treatment as compared to MD and LD cohorts. This marked elevation happens to scavenge excessive superoxide generated in the body under the condition of stress. Value of SOD and CAT tends to ascend with increase in stress as they are the key antioxidant enzyme as observed in this study. Notably heightened levels of SOD and CAT were also noted in rohu reared at elevated densities within cages [21]. This phenomenon finds concurrence in analogous contexts involving blunt snout bream [46], turbot [47], common carp (*Cyprinus carpio*) [48], Nile tilapia [36], and the GIFT strain of Tilapia [49].

The first line of antioxidant defense in fish are the activities of SOD, CAT, and GPx. These enzymes exhibit heightened activity when faced with stressful conditions, serving to counteract reactive molecules and safeguard the integrity of living cells. In the event that these enzymes do not operate at an optimal level, it leads to oxidative stress, which is characterized by the generation of Malondialdehyde (MDA) [50]. MDA is a pivotal antioxidant which serves as a primary derivative of lipid peroxidation. ROS generated amidst stress conditions target the polyunsaturated fatty acids residing in cellular membranes, thereby instigating lipid peroxidation and consequent elevation of MDA levels [39]. Although our findings indicate statistically significant difference in MDA levels at various stocking densities, that difference cannot be considered significant quantitatively. Our results suggests that high stocking density may not be a catalyst for lipid peroxidation in rohu. Different previous studies have reported unaltered MDA level in largemouth bass [51], senegalese sole [39] and turbot [52] held at diverse stocking densities.

## 5. Conclusion

In brief, the outcomes of our investigation propose that medium stocking density (3.79 kg/m^3^) outperformed the high density (5.30 kg/m^3^) in different aspects of this study. Statistically, the HD treatment exhibited a reduced growth rate compared to LD and MD treatments. However, it is impractical to interpret this as advocating for medium density as optimal and dismissing high density, as the quantitative differences observed were not substantial. However, in all conditions of stocking density, nutritional quality, survival rate, immunity, and disease resistance were not compromised. The implications of the findings of this study are that the successful intensive aquaculture with high stocking density for rohu could revolutionize the aquaculture industry by enhancing production efficiency, economic viability, resource optimization, and food security while necessitating careful consideration of environmental sustainability factors. These findings could drive further advancements and improvements in aquaculture practices, benefiting both the industry and society at large. Moreover, further studies should explore rohu and other species to increase the final biomass up to 75–150 kg/m^3^ and to evaluate the expression of genes involved in fish’s stress signaling pathway. It is also suggested that brood stock of rohu should be genetically improved to obtain stress resilient fish fingerlings which can perform better at high stocking density at large scale production level.

## Supporting information

S1 File Monthly variations (Mean ± SE) in water quality parameters, output of repeated measure ANOVA indicating the effect of stocking density, months and interactive effect of stocking density and months on different parameters measured in the study. (XLSX)

## References

[1] Mohsin M, Mu Y, Mehak A, Memon AM, Noman M. Nazir K Aquaculture in Pakistan: Status, opportunities, and challenges. 2017; 46(9): 1872–1878.

[2] Van AR, Espinoza CF, Japp D, Valderrama D, Gopal KK, Lengyel P, et al. World review of capture fisheries and aquaculture insurance. Food and Agriculture Organization. 2022 (682).

[3] Ackefors H, Huner JV, Konikoff M. Introduction to the general principles of aquaculture. CRC Press 2017.

[4] Li W, Cheng X, Xie J, Wang Z, Yu D. Hydrodynamics of an in-pond raceway system with an aeration plug-flow device for application in aquaculture: an experimental study. R. Soc. Open Sci. 2019; 6(7): 182061. 10.1098/rsos.182061 PMC6689605 31417696

[5] Fatima S, Komal W, Manzoor F, Latif AA, Liaqat R, Ameen S, et al. Analysis of the growth performance, stress, profile of fatty acids and amino acids, and cortisol in Tilapia (*Oreochromis niloticus*), cultured at high stocking density using an in-pond raceway system. Saudi J. Biol. Sci. 2021; 28(12): 7422–7431. 34867046 10.1016/j.sjbs.2021.08.048 PMC8626304

[6] Ali W, Fatima M, Shah SZH, Khan N. Naveed S. Black cardamom (*Amomum subulatum*) extract improves growth potential, antioxidant status, immune parameters and response to crowding stress in *Catla catla*. J. Anim. Physiol. Anim. Nutr. 2023. 10.1111/jpn.13888 37803872

[7] Wang Y, Xu G, Nie Z, Shao N, Li Q, Xu P. Growth performance of bluntnose black bream, channel catfish, yellow catfish, and largemouth bass reared in the in‐pond raceway recirculating culture system. N. Am. J. Aquac. 2019; 81(2): 153–159.

[8] Ni M, Wen H, Li J, Chi M, Bu Y, Ren Y, et al. The physiological performance and immune responses of juvenile Amur sturgeon (*Acipenser schrenckii*) to stocking density and hypoxia stress. Fish Shellfish Immunol. 2014; 36(2): 325–335. 24355406 10.1016/j.fsi.2013.12.002

[9] Naderi M, Keyvanshokooh S, Salati AP, Ghaedi A. Effects of dietary vitamin E and selenium nanoparticles supplementation on acute stress responses in rainbow trout (*Oncorhynchus mykiss*) previously subjected to chronic stress. Aquac. 2017; 473(1): 215–222.

[10] Garcia JA, Villarroel M. Effect of feed type and feeding frequency on macrophage functions in tilapia (*Oreochromis niloticus* L.). Fish Shellfish Immunol. 2009; 27(2): 325–329. 19501652 10.1016/j.fsi.2009.05.018

[11] Martinez-Porchas M, Martinez-Cordova LR, Ramos-Enriquez R. Cortisol and glucose: reliable indicators of fish stress. Pan-Am. J. Aquat. Sci. 2009; 2158–178.

[12] Ahmad I, Hamid T, Fatima M, Chand HS, Jain SK, Athar M, et al. Induction of hepatic antioxidants in freshwater catfish (*Channa punctatus Bloch*) is a biomarker of paper mill effluent exposure. Biochim. Biophys. Acta-Gen. Subj. 2000; 1523(1): 37–48. 10.1016/s0304-4165(00)00098-2 11099856

[13] Sahin K, Yazlak H, Orhan C, Tuzcu M, Akdemir F, Sahin N. The effect of lycopene on antioxidant status in rainbow trout (*Oncorhynchus mykiss*) reared under high stocking density. Aquac. 2014; 418(1): 132–138.

[14] Morcillo P, Esteban MA, Cuesta A. Heavy metals produce toxicity, oxidative stress and apoptosis in the marine teleost fish SAF-1 cell line. Chemosphere. 2016; 144(1): 225–233. doi: 10.1016/j.chemosphere.2015.08.020 26363324

[15] FAOSTAT. Food and Agriculture Organization of the United Nations. Statistical 2019.

[16] Crespi V, New M. Cultured aquatic species fact sheets. FAO 2009.

[17] Tahir I, Fatima M, Shah SZH, Khan N, Ali W. Interactive effects of temperature and protein levels on the growth performance, proximate composition, digestive enzyme activity and serum biochemistry of *Labeo rohita*. J. Anim. Physiol. Anim. Nutr. 2023. 10.1111/jpn.13902 37964722

[18] Mahanand SS, Moulick S, Rao PS. Water quality and growth of Rohu, *Labeo rohita*, in a biofloc system. J. Appl. Aquac. 2013; 25(2): 121–131.

[19] Narejo NT, Dayo A, Dars BA, Mahesar H, Laghari MY, Lashari PK. Effect of stocking density on growth and survival rate of *Labeo rohita* (Hamilton) fed with formulated feed. SURJ (Science Series). 2010; 42(1).

[20] Chattopadhyay DN, Mohapatra BC, Adhikari S, Pani KC, Jena JK, Eknath AE. Effects of stocking density of *Labeo rohita* on survival, growth, and production in cages. Aquac. Int. 2013; 21(1): 19–29.

[21] Swain HS, Das BK, Upadhyay A, Ramteke MH, Kumar V, Meena DK, et al. Stocking density mediated stress modulates growth attributes in cage-reared *Labeo rohita* (Hamilton) using a multifarious biomarker approach. Sci. Rep. 2022; 12(1): 1–14. 35701448 10.1038/s41598-022-13570-x PMC9197843

[22] Chappell J. In-Pond Raceway System Manual for Construction and Management; Principle Driven Aquaculture Production Technology. United States Soybean Export Council, USA. 2017.

[23] Walter HE. Proteases and their inhibitors. 2. 15. 2 Method with hemoglobin, casein, and azocoll as substrate. Methods of enzymatic analysis 1984; 270–277.

[24] Brune DE, Schwartz G, Eversole AG, Collier JA, Schwedler, TE. 19 Partitioned aquaculture systems. In Developments in Aquaculture and Fisheries Science 2004; 34(1): 561–584. Elsevier.

[25] Brown TW, Chappell JA, Boyd CE. A commercial-scale, in-pond raceway system for Ictalurid catfish production. Aquac. Eng. 2011; 44(3): 72–79.

[26] Conte L, Sonoda DY, Shirota R, Cyrino JEP. Productivity and economics of Nile Tilapia *Oreochromis niloticus* cage culture in South-East Brazil. J. Appl. Aquac. 2008; 20(1): 18–37.

[27] Abou Y, Fiogbe ED, Micha JC. Effects of stocking density on growth, yield, and profitability of farming Nile tilapia, *Oreochromis niloticus* L., fed Azolla diet, in earthen ponds. Aquac. Res. 2007; 38(6): 595–604.

[28] Telli GS, Ranzani-Paiva MJT, de Carla Dias D, Sussel FR, Ishikawa CM, Tachibana L. Dietary administration of *Bacillus subtilis* on hematology and non-specific immunity of Nile tilapia *Oreochromis niloticus* raised at different stocking densities. Fish Shellfish Immunol. 2014; 39(2): 305–311. 24878743 10.1016/j.fsi.2014.05.025

[29] Marengoni NG. Production of the Nile tilapia *Oreochromis niloticus* (*Chitralada strain*) reared in cages with different stocking densities. Arch. Zootec. 2006; 210(1): 127.

[30] Osofero SA, Otubusin SO, Daramola JA. Effect of stocking density on tilapia (*Oreochromis niloticus* Linnaeus 1757) growth and survival in bamboo–net cages trial. AJB. 2009; 8(7).

[31] Zhao H, Soufan O, Xia J, Tang R, Li L, Li D. Transcriptome and physiological analysis reveal alterations in muscle metabolisms and immune responses of grass carp (*Ctenopharyngodon idellus*) cultured at different stocking densities. Aquac. 2019; 503(1): 186–197.

[32] Memon NN, Talpur FN, Bhanger MI. A comparison of proximate composition and fatty acid profile of Indus River fish species. Int. J. Food Prop. 2010; 13(2): 328–337.

[33] Henderson RJ, Tocher DR. The lipid composition and biochemistry of freshwater fish. Prog. Lipid Res. 1987; 26(4): 281–347. doi: 10.1016/0163-7827(87)90002-6 3324105

[34] Yuan J, Ni M, Liu M, Wang H, Zhang C, Mi G, et al. Analysis of the growth performances, muscle quality, blood biochemistry, and antioxidant status of *Micropterus salmoides* farmed in in-pond raceway systems versus usual-pond systems. Aquac. 2019; 511(1): 734241.

[35] FAO. Fats and fatty acids in human nutrition. Report of an expert consultation. Food and Agriculture Organization of the United Nations 2010. 21812367

[36] Liu G, Ye Z, Liu D, Zhao J, Sivaramasamy E, Deng Y, et al. Influence of stocking density on growth, digestive enzyme activities, immune responses, antioxidant of *Oreochromis niloticus* fingerlings in biofloc systems. Fish Shellfish Immunol. 2018; 81(1): 416–422. 30056209 10.1016/j.fsi.2018.07.047

[37] Ni M, Liu M, Lou J, Mi G, Yuan J, Gu Z. Stocking density alters growth performance, plasma biochemistry, digestive enzymes, immune response, and muscle quality of largemouth bass (Micropterus salmoides) in an in-pond raceway system. Fish Physiol. Biochem. 2021; 47(1): 1243–1255. 34226986 10.1007/s10695-021-00948-3

[38] Wang Y, Xu P, Nie Z, Li Q, Shao N, Xu G. Growth, digestive enzymes activities, plasma biochemical parameters, and antioxidant status of juvenile genetically improved farmed tilapia (Oreochromis niloticus) reared at different stocking densities in an in‐pond raceway recirculating culture system. Aquac. Res. 2019; 50(4): 1338–1347.

[39] Andrade T, Afonso A, Perez-Jimenez A, Oliva-Teles A, de las Heras V, Mancera JM, et al. Evaluation of different stocking densities in a Senegalese sole (Solea senegalensis) farm: implications for growth, humoral immune parameters, and oxidative status. Aquac. 2015; 438(1): 6–11.

[40] Hastuti S, Subandiyono S, Windarto S. Blood performance of jaundice catfish *Clarias gariepinus*. Aquac. Aquar. Conserv. Legis. 2019;12(2): 480–489.

[41] Yarahmadi P, Miandare HK, Fayaz S, Caipang CMA. Increased stocking density causes changes in the expression of selected stress-and immune-related genes, humoral innate immune parameters, and stress responses of rainbow trout (*Oncorhynchus mykiss*). Fish Shellfish Immunol. 2016; 48(1): 43–53. 26549176 10.1016/j.fsi.2015.11.007

[42] Costas B, Aragao C, Dias J, Afonso A, Conceiçao LE. Interactive effects of a high-quality protein diet and high stocking density on the stress response and some innate immune parameters of Senegalese sole (*Solea senegalensis*). Fish Physiol. Biochem. 2013; 39(1): 1141–1151. 23341074 10.1007/s10695-013-9770-1

[43] Refaey MM, Li D, Tian X, Zhang Z, Zhang X, Li L, et al. High stocking density alters growth performance, blood biochemistry, intestinal histology, and muscle quality of channel catfish *Ictalurus punctatus*. Aquac. 2018; 492(1):73–81.

[44] Halliwell B, Gutteridge JM. Free radicals in biology and medicine. Oxford university press, USA 2015.

[45] Valko M, Leibfritz D, Moncol J, Cronin MT, Mazur M, Telser J. Free radicals and antioxidants in normal physiological functions and human disease. Int. J. Biochem. Cell Bio. 2007; 39(1): 44–84. 10.1016/j.biocel.2006.07.001 16978905

[46] Yang Z, Xu G, Ge X, Liu B, Xu P, Song C, et al. The effects of crowding stress on the growth, physiological response, and gene expression of the Nrf2-Keap1 signaling pathway in blunt snout bream (*Megalobrama amblycephala*) reared under in-pond raceway conditions. Comp. Biochem. Physiol. Part A Mol. Integr. Physiol. 2019; 231(1): 19–29. 10.1016/j.cbpa.2019.01.006 30641189

[47] Jia R, Han C, Lei JL, Liu BL, Huang B, Huo HH, Yin ST. Effects of nitrite exposure on hematological parameters, oxidative stress and apoptosis in juvenile turbot (*Scophthalmus maximus*). Aquat. Toxicol. 2015; 169(1): 1–9. 26476021 10.1016/j.aquatox.2015.09.016

[48] Adineh H, Naderi M, Jafaryan H, Khademi HM, Yousefi M, Ahmadifar E. Effect of Stocking Density and Dietary Protein Level in Biofloc System on the Growth, Digestive and Antioxidant Enzyme Activities, Health, and Resistance to Acute Crowding Stress in Juvenile Common Carp (*Cyprinus carpio*). Aquac. Nutr. 2022. 10.1155/2022/9344478 PMC9973225 36860436

[49] Haridas H, Verma AK, Rathore G, Prakash C, Sawant PB, Babitha RAM. Enhanced growth and the immuno‐physiological response of Genetically Improved Farmed Tilapia in indoor biofloc units at different stocking densities. Aquac. Res. 2017; 48(8): 4346–4355.

[50] Fazelan Z, Vatnikov YA, Kulikov EV, Plushikov VG, Yousefi M. Effects of dietary ginger (*Zingiber officinale*) administration on growth performance and stress, immunological, and antioxidant responses of common carp (*Cyprinus carpio*) reared under high stocking density. Aquac. 2020; 518(1), 734833.

[51] Wang Y, Xu G, Nie Z, Li Q, Shao N, Xu P. Effect of stocking density on growth, serum biochemical parameters, digestive enzymes activity and antioxidant status of largemouth bass, *Micropterus salmoides*. Pak. J. Zool. 2019; 51(1), 1509–1517.

[52] Liu B, Jia R, Han C, Huang B, Lei JL. Effects of stocking density on antioxidant status, metabolism and immune response in juvenile turbot (*Scophthalmus maximus*). Comp. Biochem. Physiol. Part—C: Toxicol. Pharmacol. 2016; 190(1), 1–8. 10.1016/j.cbpc.2016.07.007 27497046

